# Expression of a novel class of bacterial Ig-like proteins is required for IncHI plasmid conjugation

**DOI:** 10.1371/journal.pgen.1008399

**Published:** 2019-09-17

**Authors:** Mário Hüttener, Alejandro Prieto, Sonia Aznar, Manuel Bernabeu, Estibaliz Glaría, Annabel F. Valledor, Sonia Paytubi, Susana Merino, Joan Tomás, Antonio Juárez

**Affiliations:** 1 Department of Genetics, Microbiology and Statistics, University of Barcelona, Barcelona, Spain; 2 Department of Cell Biology, Physiology and Immunology, University of Barcelona, Barcelona, Spain; 3 Institute for Bioengineering of Catalonia, The Barcelona Institute of Science and Technology, Barcelona, Spain; Uppsala University, SWEDEN

## Abstract

Antimicrobial resistance (AMR) is currently one of the most important challenges to the treatment of bacterial infections. A critical issue to combat AMR is to restrict its spread. In several instances, bacterial plasmids are involved in the global spread of AMR. Plasmids belonging to the incompatibility group (Inc)HI are widespread in *Enterobacteriaceae* and most of them express multiple antibiotic resistance determinants. They play a relevant role in the recent spread of colistin resistance. We present in this report novel findings regarding IncHI plasmid conjugation. Conjugative transfer in liquid medium of an IncHI plasmid requires expression of a plasmid-encoded, large-molecular-mass protein that contains an Ig-like domain. The protein, termed RSP, is encoded by a gene (ORF *R0009*) that maps in the Tra2 region of the IncHI1 R27 plasmid. The RSP protein is exported outside the cell by using the plasmid-encoded type IV secretion system that is also used for its transmission to new cells. Expression of the protein reduces cell motility and enables plasmid conjugation. Flagella are one of the cellular targets of the RSP protein. The RSP protein is required for a high rate of plasmid transfer in both flagellated and nonflagellated *Salmonella* cells. This effect suggests that RSP interacts with other cellular structures as well as with flagella. These unidentified interactions must facilitate mating pair formation and, hence, facilitate IncHI plasmid conjugation. Due to its location on the outer surfaces of the bacterial cell, targeting the RSP protein could be a means of controlling IncHI plasmid conjugation in natural environments or of combatting infections caused by AMR enterobacteria that harbor IncHI plasmids.

## Author summary

Dissemination of antimicrobial resistance (AMR) among different bacterial populations occurs due to mainly the presence of plasmids that encode AMR determinants. IncHI plasmids are one of the groups of bacterial plasmids that confer AMR to several enterobacteria. Recently, resistance to one of the last-resort antibiotics (colistin) for some multidrug-resistant infections has spread very rapidly. IncHI plasmids represent 20% of all plasmids transmitting colistin resistance worldwide and 40% in Europe. When analyzing the interactions of the IncHI1 plasmid R27 with *Salmonella*, we identified a large-molecular-mass protein that is encoded by this plasmid and is exported to the external medium. The R27 plasmid gene coding for that protein (*R0009*) is widespread among IncHI plasmids. In this report, we characterize the protein, termed RSP. The presented data show that RSP plays a relevant role in IncHI plasmid conjugation and suggest that the protein is retained on the outer surface of the bacterial cells and facilitates cell-to-cell contact before plasmid DNA transfer. Considering that IncHI plasmids significantly contribute to AMR dissemination within enterobacteria, the findings reported in this paper suggest that the identified protein can be a target to control both IncHI-mediated AMR dissemination and infections caused by AMR enterobacteria that harbor these plasmids.

## Introduction

Infectious diseases, despite the availability of antibiotics, remain an important public health issue, representing the second leading cause of death worldwide [1]. Antimicrobial resistance (AMR) is in several instances underlying the evolution of fatal bacterial infections. The gradual increase in resistance rates of several important pathogens represents a serious threat to public health [2–4]. The dissemination of antibiotic resistance in gram-negative bacteria has been largely attributed to the acquisition of plasmid-located antibiotic resistance genes [5–7] by horizontal gene transfer (HGT).

Plasmids belonging to the incompatibility group (Inc) HI include mainly genetic elements encoding AMR determinants [8] and are widespread in *Enterobacteriaceae*. Based on the degree of DNA homology, IncHI plasmids have been classically divided into three subgroups, IncHI1, IncHI2 and IncHI3 [9], but two new Inc groups (IncHI4 and IncHI5) have been recently described [10]. Regulation of conjugative transfer of IncHI plasmids shows a distinctive feature: transfer is repressed at temperatures encountered within a warm-blooded host (37°C) and induced at temperatures found outside the host (22–30°C) [11]. Within the genus *Salmonella*, IncHI plasmids account for a significant proportion of antibiotic resistance phenotypes in the most common invasive *Salmonella* serovars: *S*. *enterica* serovar Typhi and *S*. Paratyphi A [12]. A search for IncHI plasmids within *S*. Typhi strains has shown that more than 40% of all isolates harbor an IncHI plasmid [13]. A recent study has also shown that IncHI2 plasmids predominate in antibiotic-resistant *Salmonella* isolates [14]. IncHI-encoded AMR can also be present in other enterobacterial genera, such as *Klebsiella pneumoniae* [15] and *Citrobacter freundii* [16].

Over the last three years, a novel role of IncHI2 plasmids in AMR spread has been reported. The emergence of AMR gram-negative bacteria, especially those producing carbapenemases, reintroduced colistin as a last resort antibiotic for the treatment of severe infections [17]. In contrast to its limited use in humans, colistin is widely used in food-producing animals [18]. In the past, colistin resistance was associated with chromosomal mutations only [19]. Nevertheless, plasmid-mediated resistance conferred by a mobilized colistin resistance gene (*mcr-1*) emerged recently. Since its discovery in 2016 in China [20], *mcr* genes, including the *mcr-1*/*2*/*3*/*4*/*5* variants, have been detected in bacterial organisms from human and animal microbiota, including clinical specimens and food samples in over thirty countries [21–25]. IncHI2 plasmids represented 20.5% of all plasmids encoding the *mcr-1* gene worldwide, but up to 41% in Europe [26]. This finding highlights the role of IncHI plasmids in the global epidemiology of AMR.

In addition to colistin resistance in the *Enterobacteriaceae*, IncHI2 plasmids have also been shown to encode fluoroquinolone resistance determinants in *Salmonella* [27]. Of special concern is the additionally present *mcr-1* resistance determinant in *Enterobacteriaceae* carrying carbapenem resistance genes, such as *bla*NDM and *bla*KPC. The combination of these AMR determinants will seriously compromise the treatment of infections caused by pathogenic strains harboring these plasmids [28,29]. An AMR clone of the highly virulent *E*. *coli* ST95 lineage has been recently described [30]. *E*. *coli* ST95 isolates are causative agents of extraintestinal infections, such as neonatal meningitis and sepsis. They are usually sensitive to several antibiotics. The characterized clone harbors an IncHI2 plasmid that encodes, among others, resistance determinants to colistin and several other antibiotics, including the extended-spectrum beta-lactamase blaCTX-M-1. The spread of such a clone could be a global threat to human health [30].

The plasmid R27 is the prototype of IncHI1 plasmids. It harbors the Tn*10* transposon, which confers resistance to tetracycline (Tc), and has been exhaustively studied for over 25 years. R27 replication and conjugation determinants are well characterized [31–33], and its complete nucleotide sequence is available [34]. Several ORFs from R27 (66%) do not show similarity to any known ORFs. IncHI plasmids share a common core of approximately 160 kb. The differences in size are due to the distinct presence of insertion elements. Many of them encode AMR determinants [35].

The immunoglobulin (Ig)-like domain can be identified in a large number of proteins with diverse biological functions, is widely distributed in nature, and is present in vertebrates, invertebrates, plants, fungi, parasites, bacteria, and viruses [36]. The structural feature of Ig-like domains is the presence of chains of approximately 70–100 amino acid residues present in anti-parallel β-strands and organized in two β-sheets that are packed against each other in a β-sandwich. Ig-like domains are widely distributed in bacteria. Bacterial proteins that contain an Ig-like domain (Big) are involved in several functions, such as conjugative transfer, adhesion and biofilm development [37].

We present in this report the identification and characterization of a novel protein containing an Ig-like domain that is encoded by IncHI plasmids and that, among other functions, plays a key role in plasmid conjugation. This newly identified protein may be of interest to develop new approaches to combat IncHI-mediated AMR.

## Results

### The RSP protein contains an Ig-like domain and is encoded by IncHI1 and IncHI2 plasmids

**Fig 1:**
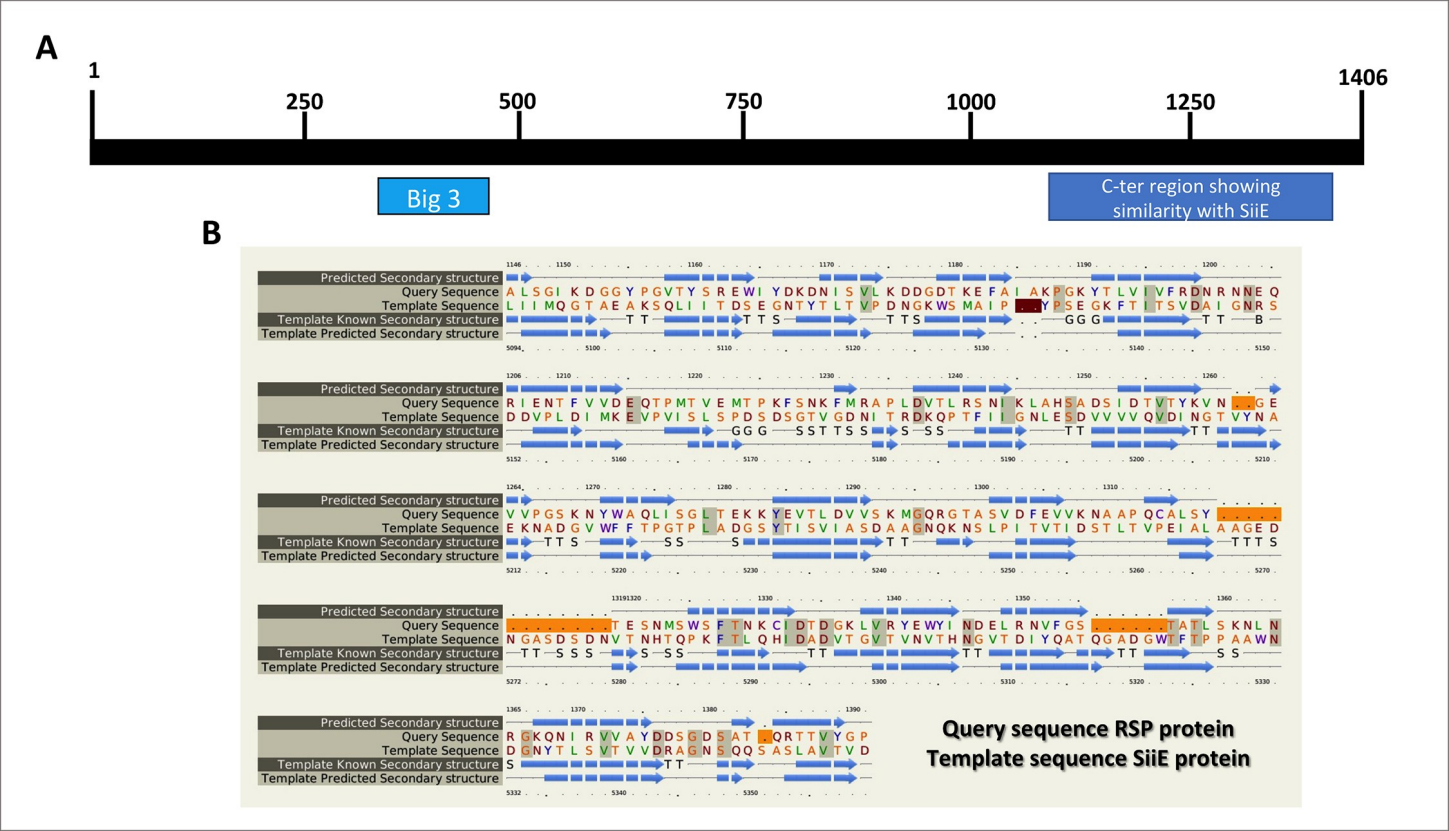
**Diagram representing the RSP protein (A) and the alignment of the C-terminal region from the RSP protein that shows similarity to the SiiE protein from *Salmonella* Thyphimurium (B).** A) The putative bacterial Ig-like domain (Big 3) that maps between the 331 and 470 amino acid residues was identified using the Conserved Domain algorithm (https://www.ncbi.nlm.nih.gov/cdd/) and the C-terminal region identified using Phyre2 program that shows similarity to SiiE are shown. B) Alignment of the C-terminal region encompassing amino acids 1146 to 1391 from the RSP protein that shows similarity to the SiiE protein from *Salmonella* Thyphimurium. The alignment was generated using the Phyre2 program. The query sequence is the RSP protein, and the template sequence is the SiiE protein, as indicated in the figure.

In a previous report, we analyzed the secretome of the *Salmonella* strain SL1344 harboring the IncHI1 plasmid R27. A large molecular mass protein (155.4 kDa) could be detected in the cell-free supernatant fraction [38]. Protein identification by LC-MS/MS analysis showed that it corresponds to an R27-encoded gene product, the product of ORF *R0009*. The R27 *R0009* gene is one of the several R27 genes whose gene products previously had no function assigned. In this report, we have addressed the characterization of the *R0009* gene product. From here on, we will term the *R0009* gene product RSP (R27-secreted protein) and the *R0009* gene *rsp*. To begin with the characterization of the protein, we first performed an *in silico* analysis by using the Phyre2 and PSIPRED secondary structure prediction algorithms (S1 Fig). The structure predicted by PSIPRED shows that the RSP protein contains only 1% alpha helices and is formed by mainly β-sheets (61%). The use of the Conserved Domain algorithm (see Material and Methods) to identify conserved domains in the RSP protein led to the identification of a group 3 bacterial Ig-like domain (Big 3) (Fig 1). The Phyre2 algorithm supports the PSIPRED prediction and shows that the C-terminal region of the RSP protein exhibits significant similarity with, among others, two large molecular mass bacterial proteins that exhibit adhesion properties (i.e., the *Staphylococcus aureus* SraP protein [39] and the *Salmonella* giant adhesion protein SiiE [40]) (S2 Fig and Fig 1). A BLASTn search of the NCBI plasmid database using the R27 *R0009* sequence showed that genes encoding RSP-like proteins are present in both IncHI1 (99% identity) and IncHI2 plasmids (87%-74% identity) (S1 Table). All 34 different IncHI1 plasmids and 102 different IncHI2 plasmids included in the NCBI database contain an *R0009* allele.

### Immunodetection of RSP in the different *Salmonella* cellular compartments

**Fig 2:**
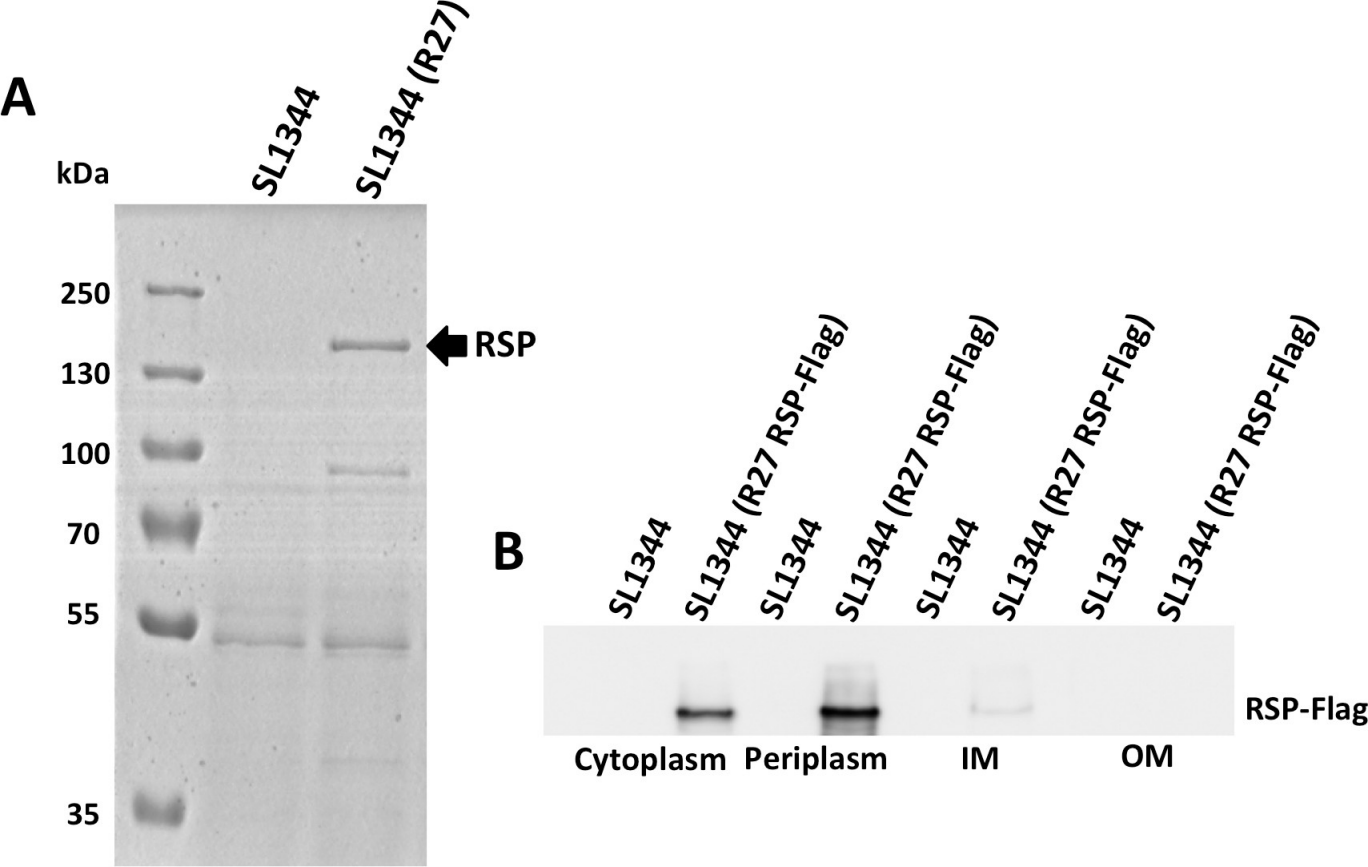
Subcellular localization of the RSP protein. A) Detection of the RSP protein in the secretome of the strains SL1344 and SL1344 (R27). The arrow points to the RSP protein. B) Immunodetection of the Flag-tagged RSP protein with anti-Flag antibodies in the different cellular compartments. RSP-Flag is indicated. Experiments were repeated three times. A representative experiment is shown.

To identify the RSP protein in the different cellular compartments, a Flag tag was added to the *rsp* gene (see materials and methods section for details). Cultures of the strain SL1344 (R27 RSP-Flag) were grown in LB medium at 25°C to an O.D._600 nm_ of 2.0. Samples were then collected, and the different cellular fractions were obtained. RSP was detected by western blotting, using anti-Flag antibodies (Fig 2). The protein was detected in the periplasm, inner membrane and cytoplasmic fractions. The presence of this protein in different envelope compartments of the cell suggests that RSP can be translocated to the outer surface of the cell. Therefore, it could be identified when the secretome was analyzed.

### The RSP protein is required for R27 plasmid conjugation

**Fig 3:**
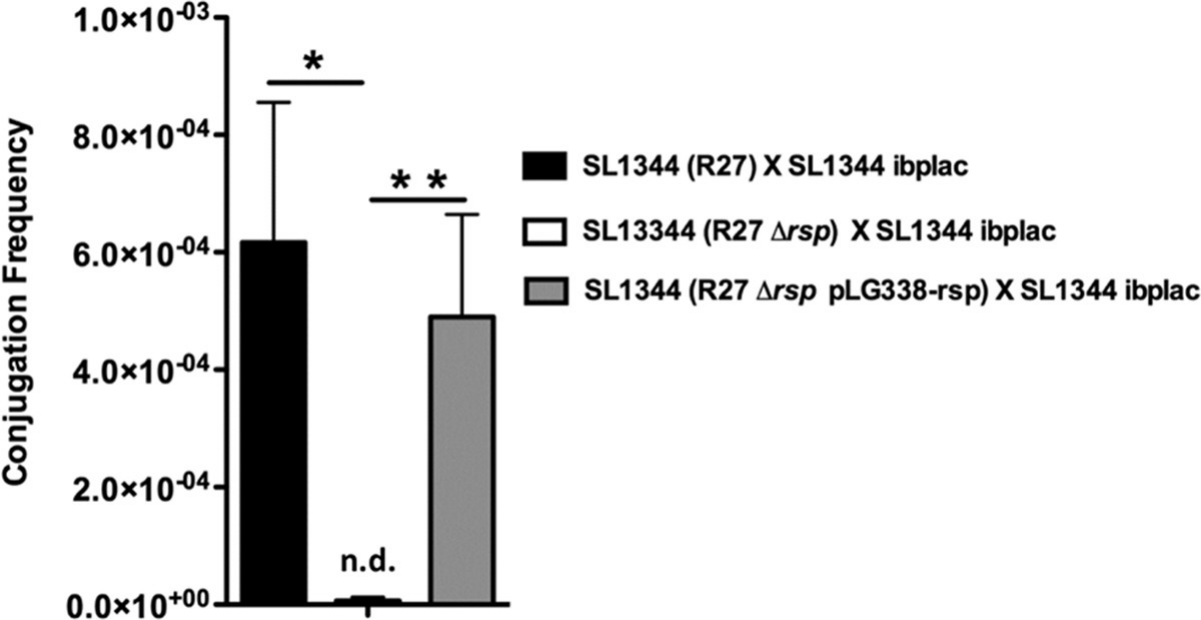
The RSP protein is required for conjugation of the R27 plasmid. Transfer frequencies of the R27 and R27 Δ*rsp* plasmids. The recipient strain was the SL1344 ibplac strain. The donor strains were SL1344 (R27), SL1344 (R27 Δ*rsp*) and SL1344 (R27 Δ*rsp* pLG338-rsp). The data shown are the means and standard deviations of three independent experiments. Statistical analysis showed a significant difference (*P-value < 0.001; **P-value < 0.005). n.d., not detectable.

*rsp* maps in one of the R27 regions required for plasmid transfer, the Tra2 region [32,41] (S3 Fig). In a previous study [32], the *rsp* gene was disrupted by inserting a chloramphenicol resistance cassette, and its effect on the conjugation frequency of an R27 mutant derivative (drR27) that exhibits a conjugation frequency that is higher than that of the wt plasmid was determined. *rsp* mutants exhibited a reduced conjugation frequency of the drR27 plasmid, but complementation experiments were not performed. We therefore decided to clarify the role of the RSP protein in wt R27 plasmid conjugation. Upon constructing an R27 derivative lacking the *rsp* gene (the plasmid R27 Δ*rsp*), the conjugation frequency of the strains SL1344 (R27) and SL1344 (R27 Δ*rsp*) growing at 25°C was compared. In three independent experiments, transfer of the R27 Δ*rsp* plasmid could not be detected at a frequency higher than 3 x 10^−7^ (Fig 3). To correlate RSP loss with the observed effect on R27 plasmid conjugation, we cloned the *rsp* gene with its own promoter in the low copy number vector pLG338-30, obtaining the plasmid pLG338-rsp. Transformation of this latter plasmid in the strain SL1344 (R27 Δ*rsp*) resulted in R27 Δ*rsp* transfer at a frequency only slightly lower than that of wt R27 (Fig 3).

### Temperature and growth medium influence RSP transcription

**Fig 4:**
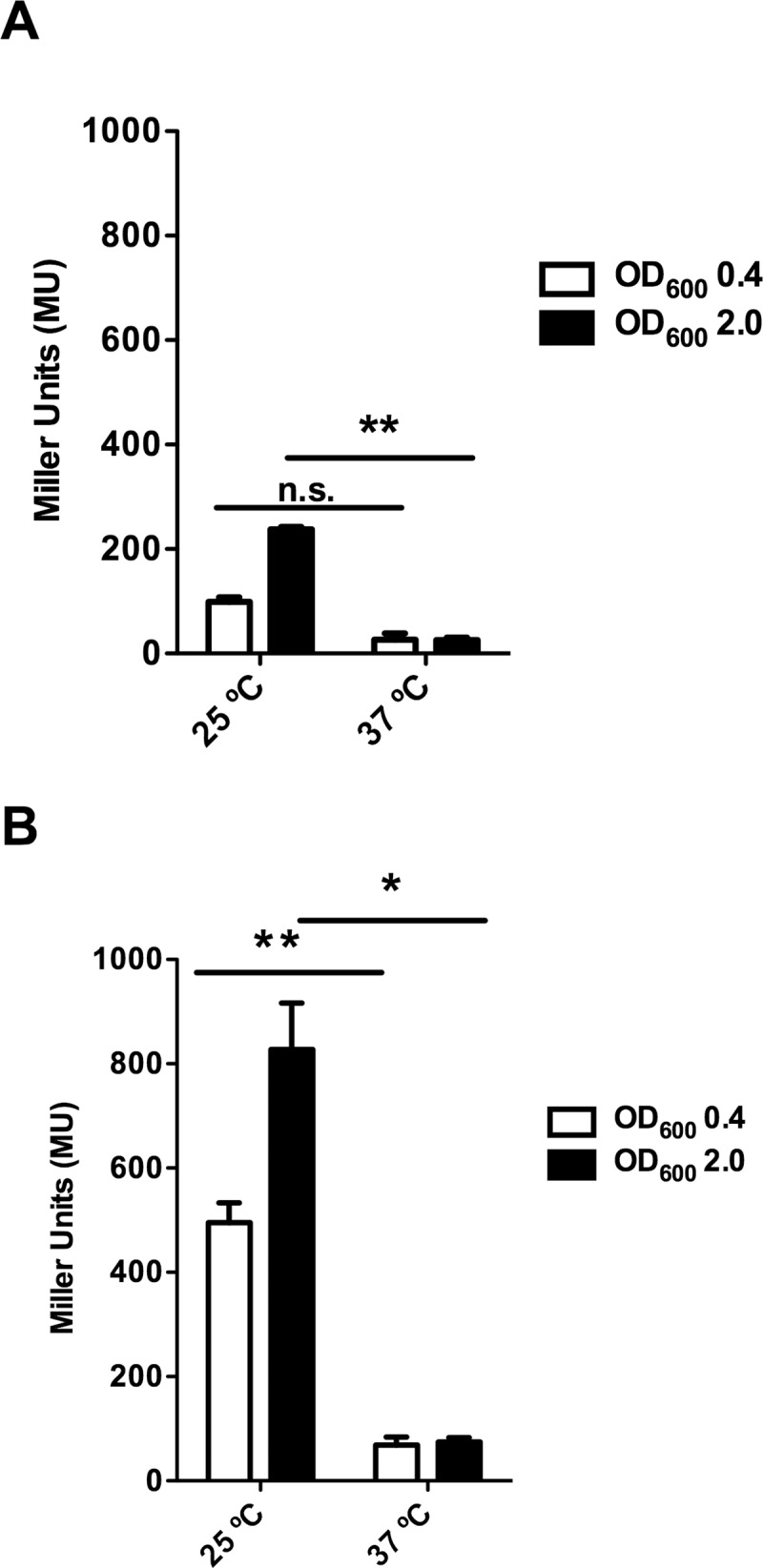
Temperature- and growth medium-dependent expression of the *rsp* gene. Effect of the growth medium and growth temperature on transcription of the *rsp* gene determined by using a *rsp*::*lacZ* transcriptional fusion. Samples were collected from cultures grown in LB (A) or M9 media (B) at 25 and 37°C., either at the exponential (O.D._600 nm_ 0.4) or early stationary (O.D._600 nm_ 2.0) growth phases. β-galactosidase activity is expressed as Miller units. The data shown are the means and standard deviations of three independent experiments. Statistical analysis showed a significant difference (*P-value < 0.001, **P-value < 0.005). n.s., not significant.

Previous reports suggested that the transcription of genes mapping in the R27 Tra2 region is thermoregulated [11,33]. We decided both to confirm RSP thermoregulation and to assess the effect of the growth medium on RSP expression. To that end, we constructed an *rsp*::*lacZ* transcriptional fusion and measured *rsp* transcription at 25°C and 37°C both in rich (LB) and minimal (M9) media. Samples were both collected at the exponential and early stationary growth phases. In accordance with the observed effect of temperature on IncHI plasmid conjugation, low temperature influences *rsp* transcription, and this effect occurs in both culture media used (Fig 4). Nevertheless, we could also observe that when comparing cultures grown in both media, *rsp* transcription is significantly higher in cells grown in minimal medium than in cells grown in LB medium. In fact, *rsp* transcription at 37°C in M9 medium is only four times lower than *rsp* transcription at 25°C in cells growing in LB medium (Fig 4). We also used qRT-PCR to assess the effect of the growth temperature on *rsp* transcription in cells grown at 25 and 37°C in LB medium until the onset of stationary phase (O.D._600 nm_ of 2.0). Transcription of the *rsp* gene is several orders of magnitude (more than 40-fold) lower at 37°C than at 25°C.

### Expression of the RSP protein reduces motility

**Fig 5:**
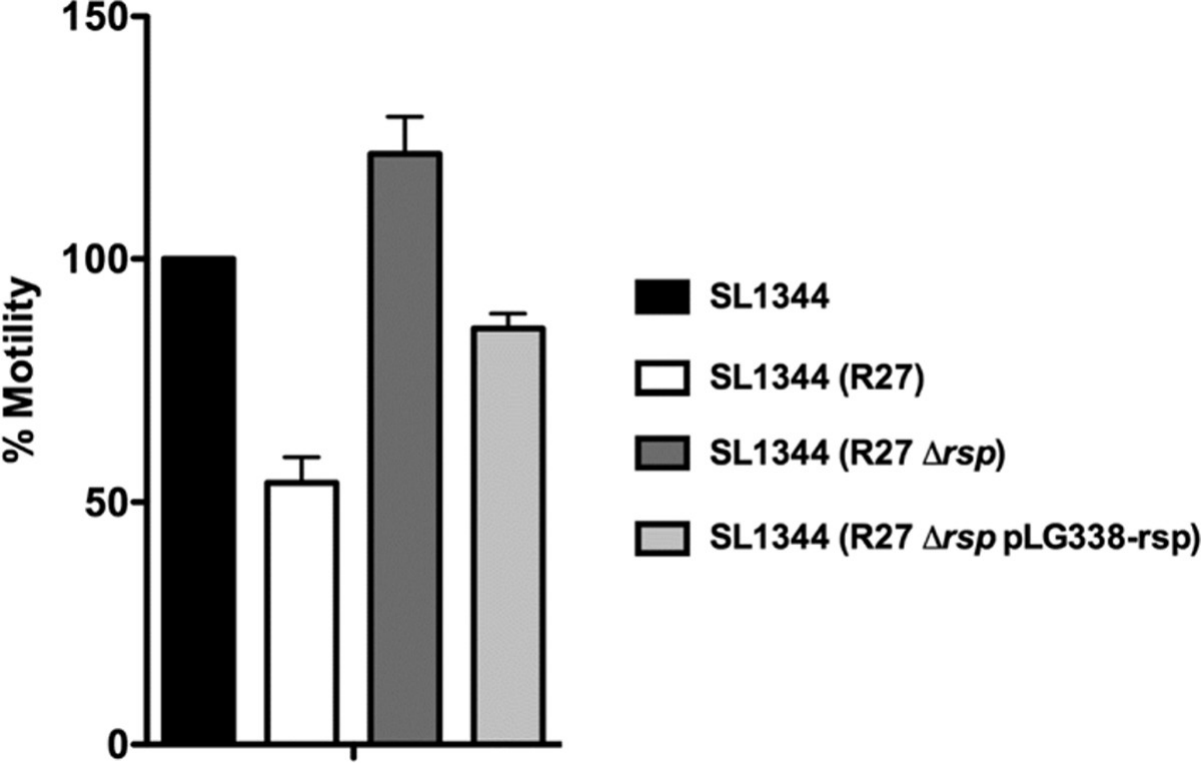
Expression of the RSP protein reduces cell motility. Relative motility of the strains SL1344, SL1344 (R27), SL1344 (R27 Δ*rsp*) and SL1344 (R27 Δ*rsp* pLG338-rsp). Motility of the strain SL1344 (measured as the diameter of the growth zone in the agar plate) was considered as 100%. The results are the means of three independent experiments. Standard deviations are shown.

We previously showed that acquisition of the R27 plasmid by *Salmonella* results in reduced motility [42]. Considering that the RSP protein is exported to the external surface of the cell and that it influences conjugation, we decided to determine whether this protein might play a role in the observed R27-dependent reduced motility of *Salmonella* cells. To assess this possibility, we performed a comparative motility assay with the *Salmonella* strain SL1344 and its derivatives incorporating the R27, R27 Δ*rsp*, and R27 Δ*rsp* pLG338-rsp plasmids. The results obtained (Fig 5, S4 Fig) show that R27-dependent motility loss requires the synthesis of the RSP protein. This phenotype can be complemented by providing the RSP protein *in trans*: the comparatively increased motility that is observed in the strain R27 Δ*rsp* is reduced when the plasmid pLG338-rsp is incorporated (Fig 5).

### The *trhC* gene from the R27-encoded type IV secretion system is required for RSP export

**Fig 6:**
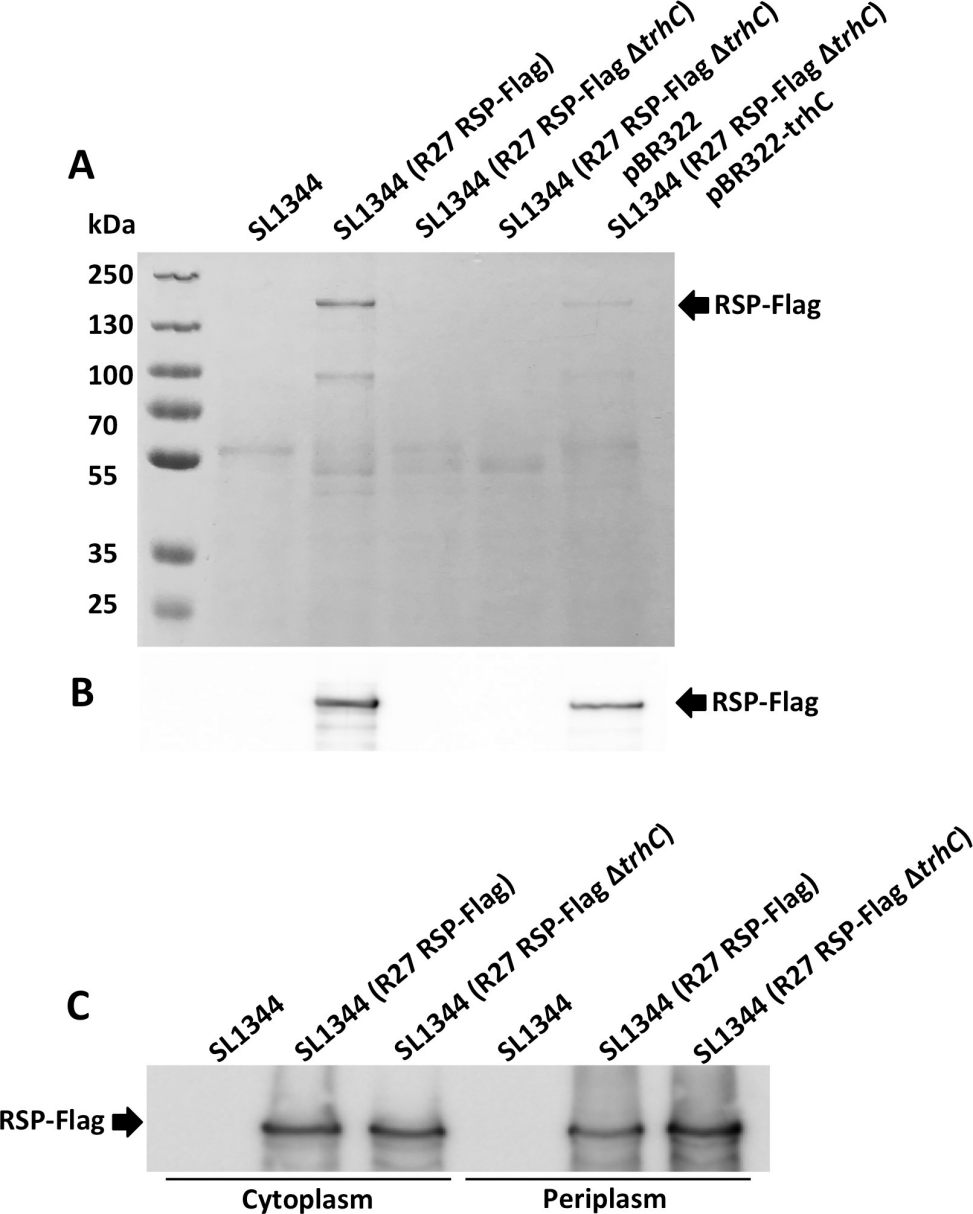
The R27 T4SS *trhC* ATPase is required for RSP export. A) SDS-PAGE analysis of the secretomes of different constructs of the strain SL1344 containing different R27 derivatives. Coomassie blue staining (A) and immunostaining with Flag-specific antibodies (B). C) Immunodetection of the RSP protein in the cytoplasm and periplasmic fractions of the different constructs. Inactivation of the R27 *thrC* gene interferes with RSP export in the strain SL1344 (R27). RSP export is restored in the strain SL1344 (R27 Δ*trh*C) by providing *in trans* the plasmid pBR322-*thrC*. The arrow points to the RSP protein. Experiments were performed three times. A representative experiment is shown.

We next studied the mechanism by which the RSP protein is exported. To address this point, we first conjugated the R27 plasmid to the *E*. *coli* strain MG1655 and analyzed the secretome of the transconjugants. The RSP protein could be detected by SDS-PAGE as it has been detected in *Salmonella* (S5 Fig). This observation suggests that (i) both the strains SL1344 and MG1655 harbor chromosomally encoded secretion determinants for the RSP protein, (ii) RSP is an autotransporter and is secreted via a type V secretion system, or (iii) the R27 plasmid encodes the determinants responsible for RSP export. Considering the limited secretory ability of *E*. *coli* MG1655, it seems unlikely that this strain would express a secretion system that would account for RSP export. The use of the SSPred program (http://www.bioinformatics.org/sspred/html/sspred.html) suggested that the RSP protein could be exported through a type IV secretion system (S6 Fig). To provide evidence supporting this hypothesis, we decided to knock out the ATPase encoded by the R27 *trhC* gene [43] and check the presence of the RSP protein in the secretome of the strain SL1344 (R27 Δ*trhC*). The RSP protein could not be detected in the secretome of the strain SL1344 (R27 Δ*trhC*) but could be detected intracellularly (Fig 6). We next checked the complementation of RSP export in the strain SL1344 (R27 Δ*trhC*) by providing *in trans* the gene encoding the ATPase cloned in the plasmid pBR322 (plasmid pBR322-trhC). Complementation of RSP export could be observed (Fig 6A–6C), thus suggesting that the R27-encoded type IV secretion system mediates export of the RSP protein.

### The RSP protein is associated with the flagella synthesized by the strain SL1344

**Fig 7:**
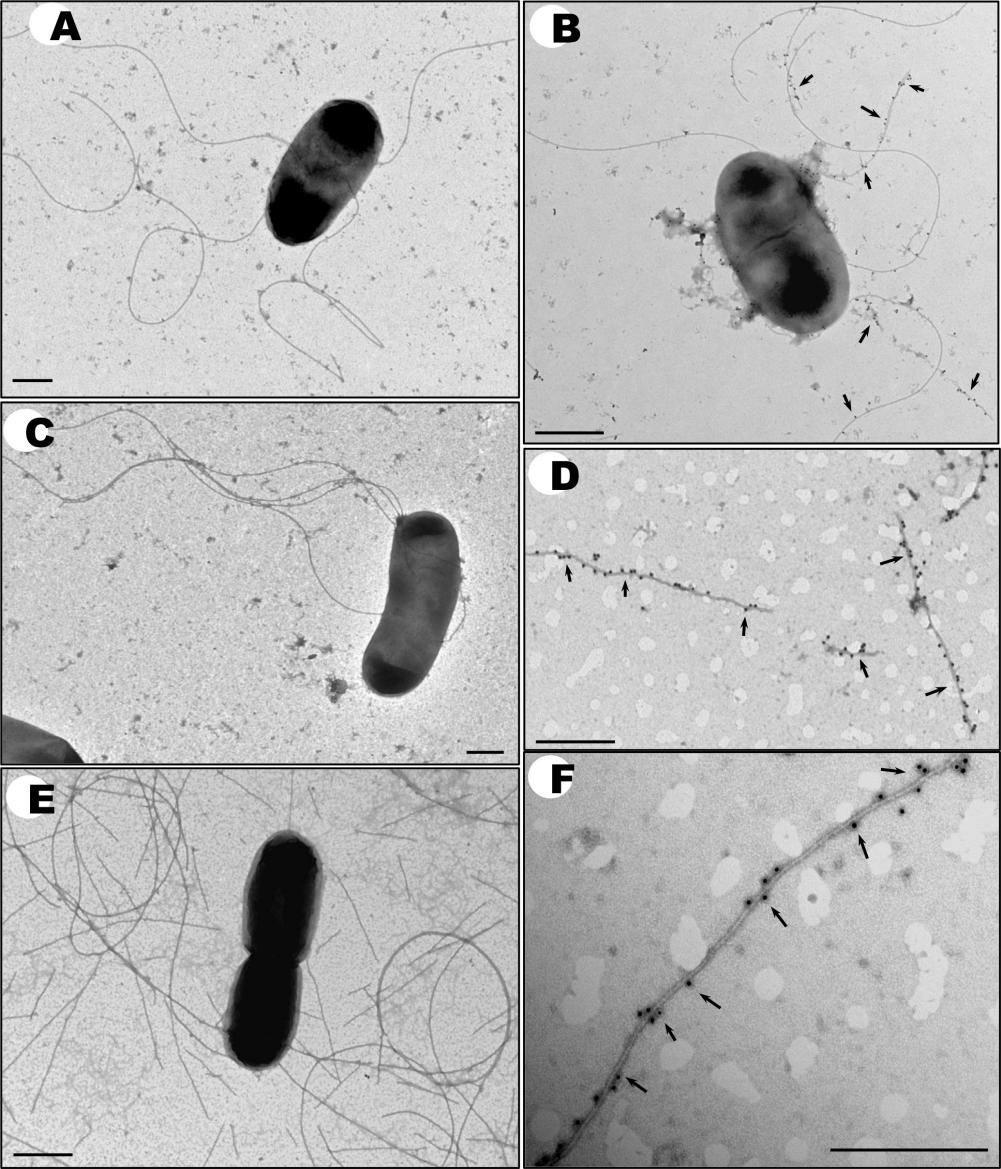
Detection of the RSP protein by immunogold electron microscopy. The plasmid-free SL1344 strain is shown in panel (A). The strain SL1344 (R27) is in panels (B, D-F). The strain SL1344 (R27Δ*rsp*) is shown in panel (C). Samples in panels (A-D and F) were labeled with rabbit anti-RSP polyclonal antibodies and goat anti-rabbit IgG conjugated to 12 nm gold particles. The control experiment is shown in panel (E) and was labeled with goat anti-rabbit IgG conjugated to 12 nm gold particles in the absence of a specific antibody. Arrows point to the RSP protein associated with the flagella. Bars represent 0,5 μm.

As shown above, the RSP protein can be detected in different cellular compartments of strain SL1344 cells but not in the outer membrane. To better understand the cellular location of RSP, we performed transmission electron microscopy studies by using gold-labeled antibodies raised against the Big 3 domain of the RSP protein. No gold particles were found to be associated with plasmid-free SL1344 cells or SL1344 (R27 Δ*rsp*) cells (Fig 7A and 7C). In contrast, gold particles could be found associated with mainly the flagellar filaments of SL1344 (R27) cells (Fig 7B). A detailed image of flagella fragments shows gold particles associated with specific structures attached to the flagella (Fig 7D and 7F). Flagella containing the RSP protein are frequently present as broken fragments. Because of the observed interaction, we studied whether the R27 protein copurifies with flagellin, and this possibility was indeed occurred (S7 Fig).

### The RSP protein, flagella and R27 conjugation

**Fig 8:**
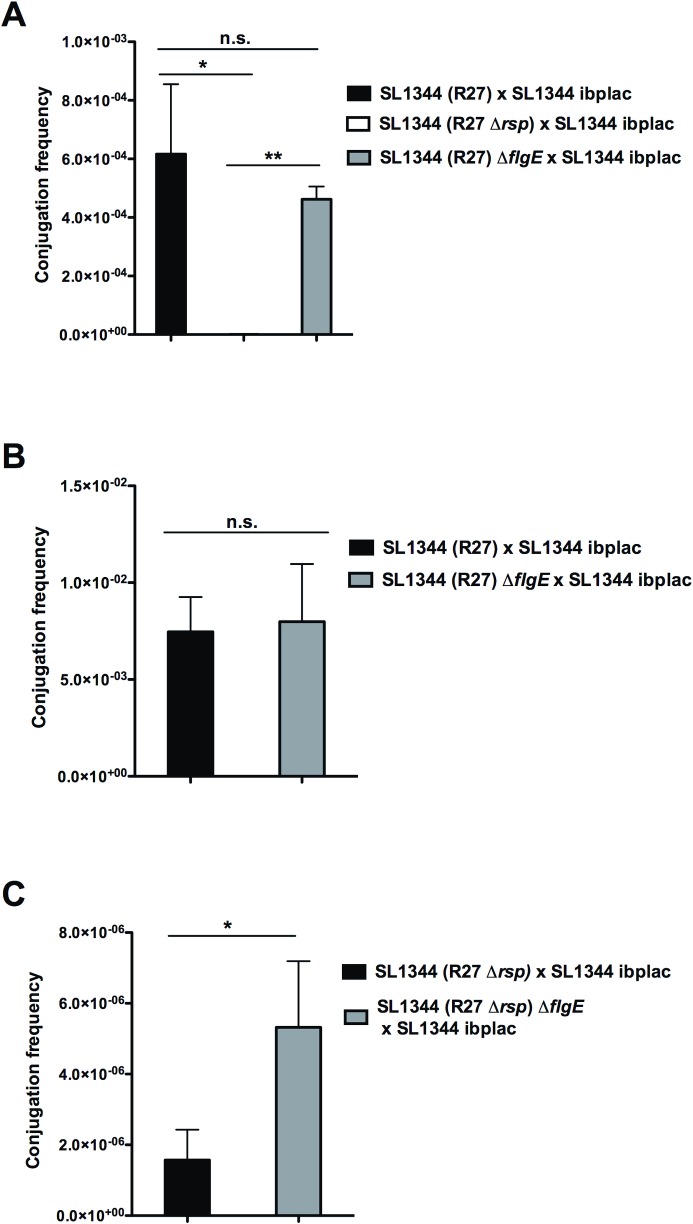
Conjugation frequencies of R27 and R27 Δ*rsp* plasmids using SL1344 and SL1344 Δ*flgE* as donor strains. Conjugations were performed either in liquid (A) or on nitrocellulose filters (B, C). The data shown are the means and standard deviations of three independent experiments. Statistical analysis showed significant differences (*P-value < 0.005; **P-value < 0.001), n.s., not significant.

Considering that the RSP protein is required for R27 plasmid conjugation and that it is associated with the flagella, we decided to analyze the role of the RSP protein in R27 conjugation in donor cells lacking flagella. To this end, we constructed an SL1344 (R27) Δ*flgE* derivative. The strain SL1344 (R27) Δ*flgE* does not synthesize the flagellar hook, it is nonmotile, and the RSP protein is expressed (S7 Fig). We assessed whether the RSP protein differentially influenced conjugation in the wt and the *flgE* derivative of the strain SL1344 (R27). We used two experimental designs: conjugation in liquid medium or on nitrocellulose filters. When conjugation was performed in liquid medium, the strain SL1344 (R27) Δ*flgE* showed a conjugation frequency similar to that of the wt strain (Fig 8A). Expression of the RSP protein was also required for plasmid conjugation in the strain SL1344 (R27) Δ*flgE* (Fig 8A). When matings took place on nitrocellulose filters, the strain SL1344 (R27) Δ*flgE* also showed a conjugation frequency similar to that of the wt strain (Fig 8B). On the other hand, by using this latter experimental approach, transconjugants could be detected at a low frequency when the strain SL1344 (R27 Δ*rsp*) was used as the donor (Fig 8C). Notably, the conjugation frequency observed in the strain SL1344 (R27 Δ*rsp*) Δ*flgE* was significantly higher than that in the strain SL1344 (R27 Δ*rsp*) (Fig 8C). Hence, loss of the RSP protein differentially influences conjugation in flagellated and nonflagellated *Salmonella* cells under specific mating conditions.

### Opsonization of the strain SL1344 (R27 RSP-Flag) with Flag-specific antibodies results in increased phagocytosis by bone marrow-derived macrophages

**Fig 9:**
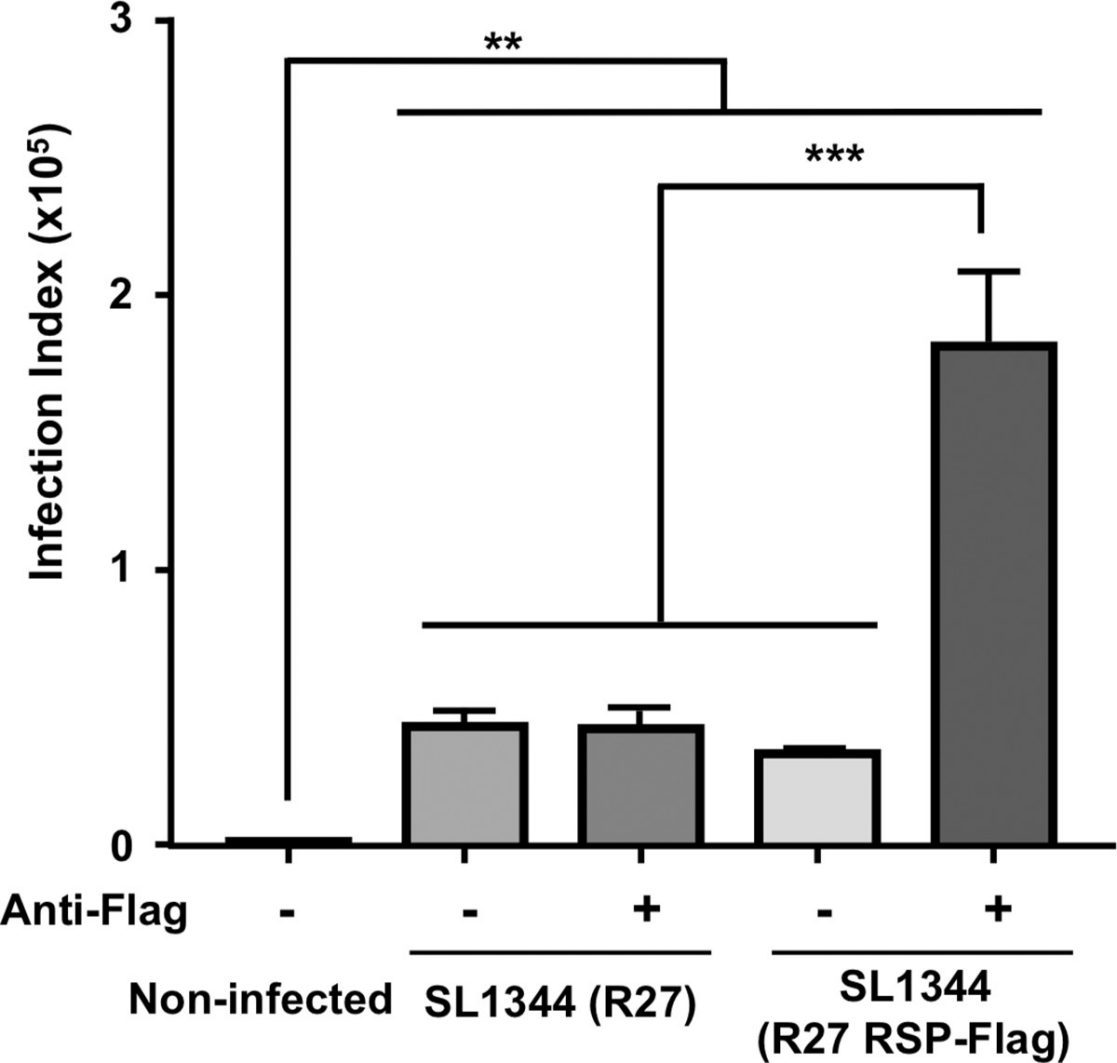
Opsonization of RSP-Flag enhances bacterial phagocytosis by macrophages. Strain SL1344 cells expressing either RSP (wild-type R27) or RSP-Flag (RSP-Flag R27) were opsonized with anti-Flag antibodies or left nonopsonized. Macrophages were infected for 30 min with *Salmonella* cells (either opsonized or nonopsonized) at an MOI of 15. Negative control cells were not infected. The results are the mean of 2 independent experiments each of which was performed using biological triplicates. One-way ANOVA, Tuckey´s post hoc (***P < 0.0001; **P < 0.01).

Considering that the RSP protein is exported to the outer surfaces of *Salmonella* cells, we decided to assess whether opsonization of SL1344 (R27 RSP-Flag) cells with anti-Flag antibodies would result in increased phagocytosis. For this purpose, bone marrow-derived macrophages (BMDMs) were exposed to *S*. *enterica* serovar Typhimurium SL1344 expressing either RSP (R27 wild type) or RSP-Flag (R27 RSP-Flag) that had previously been incubated with anti-Flag antibodies, and the levels of internalized bacteria were measured by flow cytometry. Significantly higher numbers of infected macrophages were observed after exposure to opsonized RSP-Flag-expressing bacteria than of BMDM exposed to nonopsonized bacteria (Fig 9). Thus, binding of anti-Flag antibodies to the surface of RSP-Flag increases the capability of macrophages to phagocytose bacterial cells expressing this protein, further supporting the surface localization of RSP in SL1344 cells.

## Discussion

Details of IncHI plasmid conjugation, including the effect of temperature and the role of global regulators, such as the H-NS and Hha proteins, in the thermoregulation of IncHI conjugation, have been known for several years [11,31–33,44]. Nevertheless, the role of the RSP protein had been hitherto overlooked. Our study exemplifies the relevance of assigning function to the large percentage of sequenced genes that code for proteins of unknown function, as was the case for the *rsp* gene of the plasmid R27. The fact that RSP protein homologs are encoded in all IncHI1 and IncHI2 plasmids hitherto sequenced and deposited in the NCBI database highlights the importance of RSP function in the biology of these plasmids and their hosts. By studying the RSP protein, we shed light on novel aspects of IncHI plasmid conjugation and focused on the design of a novel strategy to combat infections caused by bacteria harboring IncHI plasmids.

As suggested from the fact that the *rsp* gene maps in the Tra2 region of the R27 plasmid (S3 Fig), which includes several conjugative determinants, the RSP protein plays a relevant role in R27 conjugation. Previous studies that used a conjugation-derepressed mutant derivative of the R27 plasmid (dR27) suggested that the *rsp* gene product reduces the conjugation frequency of the plasmid [32]. By using the wt R27 plasmid here, we show that the expression of the RSP protein is required for R27 conjugation in SL1344 cells growing in liquid medium. Inactivation of the *rsp* gene reduces the conjugation frequency to experimentally undetectable values, and complementation of the *rsp* mutation by the plasmid pLG338-rsp restores the conjugation frequency observed in wild-type cells.

Semi quantitative RT-PCR analysis of transcription in the R27 Tra2 region where *rsp* maps suggested that transcription of genes mapping in Tra2 is thermoregulated [33]. Thermoregulation of IncHI plasmid transfer has long been known [45]. Classically, thermoregulation of IncHI plasmid conjugation was interpreted as a means of facilitating dissemination of antibiotic resistance determinants in natural water and soil environments [11,34,46]. Nevertheless, some studies could show that these plasmids facilitate the adaptation of enterobacteria such as *Salmonella*, to nonhost environments [42]. Incorporation of the IncHI plasmid R27 significantly impacts the *Salmonella* transcriptome when cells enter stationary phase and grow at low temperature (25°C). Optimal dissemination of IncHI plasmids at low temperatures can thus be interpreted as these plasmids favoring fitness of their bacterial hosts when they thrive in the environment or in hosts such as plants. R27 also modified the *Salmonella* transcriptome at 37°C [42], thus suggesting that the R27 plasmid can also influence *Salmonella* physiology within the host [42]. Whereas these plasmids show very reduced transfer frequencies at 37°C, the facts that they also modify the bacterial transcriptome within the host and that they encode AMR determinants [8] provide evidence for their clinical role. We confirm in this report that *rsp* transcription is thermoregulated and provide further data about the regulation of *rsp* expression. The nature of the growth medium also influences *rsp* transcription. Growth in minimal medium at high temperature allows moderately high levels of *rsp* transcription. Hence, even within the host at 37°C, significant levels of RSP protein expression can occur.

Some of the data presented in this report shed light on one of the roles of the RSP protein and hence on novel features of IncHI plasmid conjugation. The type IV secretion system encoded by the R27 plasmid likely mediates RSP export. This phenomenon has already been reported for several type IV secretion systems, which are known to transfer both proteins and relaxosomes [47–51]. The existence of a periplasmic RSP intermediate suggests that the protein is translocated by a type IV piston-like mechanism [52]. The flagellar filaments of SL1344 cells appear to be one of the targets of extracellular RSP protein. Interaction of the RSP protein with the flagellar filament may affect its structural stability. In fact, flagella interacting with the RSP protein are shorter and more breakable than those from cells not expressing the RSP protein. Although this latter observation may be a consequence of the manipulation of the bacterial cells for electron microscopy observation, it is apparent that the likely consequence of the interaction of the RSP protein with the flagella must be the alteration of flagellar function, which, in turn, leads to the observed reduced bacterial cell motility. IncHI plasmids reducing bacterial cell motility also appears to occur by another mechanism: R27 plasmid encoded regulators downregulate flagella synthesis [53]. The results presented both in this latter report and in the present paper support the view that the cell motility is reduced when IncHI plasmid conjugation is prompted. Nevertheless, we show here that motility reduction is only one of the events that promote efficient plasmid transfer: the RSP protein is also required by nonflagellated donor cells to efficiently transfer the R27 plasmid. Therefore the interaction of the RSP protein with the flagella is not the main reason why this protein plays a very important role in R27 conjugation. In spite of this, a relationship among the RSP protein, the flagella and the R27 conjugation frequency can be established when specific mating conditions take place. When the mating pairs are placed in nitrocellulose filters and *flgE* mutants are used as donors, the conjugation frequency of the plasmid R27 Δ*rsp* is more than threefold higher than that observed when *flgE* cells harboring the wt R27 plasmid are used as donors. These results show a differential effect of the RSP protein in flagellated and nonflagellated cells. The absence of the flagella reduces the impact of RSP loss on the conjugation frequency. A likely hypothesis is that the interaction of the RSP protein with flagella may reduce motility, being a first step to favor conjugation but requiring additional RSP function(s). RSP interacting with the flagella in cells grown in liquid medium is observed in most of the SL1344 (R27) cells (about 70%). Nevertheless, the R27 conjugation frequency between cells growing under these conditions is somewhat less than 10^−3^. This clearly shows that expression of conjugation functions is just a requisite for conjugation to occur, but plasmid transfer requires several concatenated events to take place. Any interference in the process (i.e., disruption of the mating pairs) may interrupt conjugation.

Adhesion is a function displayed both by several bacterial proteins containing an Ig-like domain [37] and by two other proteins whose C-terminal domains show similarity to the RSP C-terminal domain: SraP and SiiE. The *S*. *aureus* SraP protein binds to sialylated receptors on platelets and lung epithelium [39,40,54], and the *S*. *enterica* giant adhesion protein SiiE enables apical invasion into enterocytes [55]. It is therefore likely that the RSP protein may also facilitate cell-to-cell adherence, which is critical for IncHI plasmid conjugation. Hence, the RSP protein must play different roles in IncHI plasmid conjugation. By binding to flagella and subsequently reducing motility, RSP may facilitate random recipient/donor collisions, leading to cell-to-cell contact and generating mating pairs. Consolidation of the mating pairs must require the adhesion properties of RSP. Bacterial cell clumping has been shown to increase the conjugation frequency of some plasmids [56–58]. In some instances, the conjugative system encodes a clumping protein that promotes cell aggregation, which in turn results in an increased conjugation frequency [57]. Whereas large aggregates, such as those observed in these systems, are not apparent in the strain SL1344 (R27) in the presence of recipient cells, RSP function in IncHI plasmid conjugation may resemble the function of clumping proteins in other systems.

RSP-mediated adherence could also facilitate attachment to other surfaces. We show in this report that although RSP expression is thermoregulated, it can also be expressed at 37°C, with expression levels dependent on the nature of the culture medium. Taking into account the role of proteins such as SraP and SiiE in adherence to eukaryotic cells, RSP expression within the host, regardless of promoting conjugation, might also facilitate adherence of enterobacteria harboring an IncHI plasmid to specific receptors in host tissues.

Infections by AMR *Enterobacteriaceae* in immunocompromised patients and others may result in fatality [59]. The recently reported relevant role of IncHI plasmids disseminating AMR, specifically colistin resistance [22–26,30,60] highlights the urgent need to control IncHI plasmid dissemination. Considering that (i) the RSP protein is expressed by both IncHI1 and IncHI2 plasmids; (ii) the RSP protein is exported to the outer surfaces of the bacterial cell; and (iii) Flag-tagged RSP is recognized by Flag-specific antibodies, leading to opsonization and increased phagocytosis by macrophages, it is apparent that targeting the RSP protein can become a strategy for both restricting the dissemination of IncHI plasmids and combatting infections caused by enterobacterial strains harboring any of them.

Inhibiting bacterial conjugation has been suggested as an important strategy to reduce the persistence of antibiotic resistance in natural environments [7,61]. Considering that RSP loss results in IncHI plasmid conjugation inhibition, targeting of the RSP protein by nonpathogenic bacterial cells expressing RSP-directed nanobodies could represent a novel strategy focused on controlling the dissemination of IncHI plasmids in some natural environments, such as livestock farms or water treatment plants. Vaccination is one of the relevant approaches that should be fostered to combat AMR. In this context, multiantigen vaccines may favor competing bacteria in the different colonizing niches, thus reducing the incidence of AMR pathogens [62]. When proven to be antigenic, the RSP protein can be considered a candidate to be included in these vaccines.

## Materials and methods

### Ethics statement

The protocol requiring animal manipulation has been approved by the Institutional Animal Care and Use Committee (IACUC) from Parc Científic de Barcelona (PCB) (project #9672). The PCB Animal Facility is accredited and registered by the Generalitat of Catalonia government (registration # B-9900044) as a breeding and user center for laboratory animal research and for the breeding and use of genetically modified organisms (GMOs). IACUC-PCB considers that the abovementioned project complies with standard ethical regulations and meets the requirements of current applicable legislation (RD 53/2013 Council Directive; 2010/63/UE; Order 214/1997/GC).

### Bacterial strains, plasmids and growth conditions

The bacterial strains and plasmids used in this work are listed in S2 Table. The bacterial strains were routinely grown in Luria-Bertani (LB) medium (10 g l^-1^ NaCl, 10 g l^-1^ tryptone and 5 g l^-1^ yeast extract), or as indicated in the text, cells were also grown in M9 minimal medium [63] supplemented with glucose at a final concentration of 0.4% with vigorous shaking at 200 rpm (Innova 3100, New Brunswick Scientific). The antibiotics used were chloramphenicol (Cm) (25 μg ml^-1^), tetracycline (Tc) (15 μg ml^-1^), carbenicillin (Cb) (100 μg ml^-1^) and kanamycin (Km) (50 μg ml^-1^) (Sigma-Aldrich).

### Genetic manipulations

All enzymes used to perform standard molecular and genetic procedures were used according to the manufacturer’s recommendations. To introduce plasmids into *E*. *coli* and *Salmonella*, bacterial cells were grown until an O.D._600 nm_ of 0.6. Cells were then washed several times with 10% glycerol, and the respective plasmids or DNA were electroporated by using an Eppendorf gene pulser (Electroporator 2510).

Deletions of the *rsp* (ORF *R0009)*, *trhC* and *flgE* genes were performed in the strain SL1344 (R27) by using the λ Red recombination method, as previously described [64]. The antibiotic resistance determinant of the plasmid pKD3 was amplified using the corresponding oligonucleotides RS0009_P1/RS0009_P2 and trhCP1/trhCP2 for the *rsp* and *trhC* genes, respectively (S3 Table), and the resistance determinant of the plasmid pKD4 was amplified using the corresponding oligonucleotides SL1344flgEP1/SL1344flgEP2 for the *flgE* gene (S3 Table). The mutants were confirmed by PCR using the oligonucleotides RS0009_Up_for/RS0009_down_rev for *rsp*, trhCP1up/trhCP2down for *trhC* and SL1344flgEP1up.1/SL1344flgEP2down.1 for the *flgE* gene (S3 Table).

A transcriptional *lacZ* fusion was made in the *rsp* gene from the R27 plasmid. The antibiotic resistance determinant from the plasmid R27 rsp was eliminated using an FLP/FRT-mediated site-specific recombination method, as previously described [65], thus, generating the plasmid R27 Δ*rsp*. A FRT-generated site was used to integrate the plasmid pKG136 [66], thereby generating a transcriptional *lacZ* fusion.

Recombinational transfer of the Flag sequence into the *rsp* gene was achieved by following the methodology described in [67]. The template vector coding for Flag and Km^r^ used was pSUB11. The primers used for the construction of the Flag-tagged derivative were R27_p0103XP1 and R27_p0103XP2 (S3 Table). The correct insertion of the Flag-tag was confirmed by PCR using oligonucleotides R27_p0103XP1UP and R27_p0103XP2DOWN (S3 Table).

To construct the plasmids pLG338-rsp and pBR322-trhC, ORF *R0009* (*rsp*) and the *trhC* gene (GenBank accession number NC_002305.1, positions 11659–16099 and 28465–32972, for *R0009* and *trhC* genes, respectively) were amplified using the oligonucleotides 09-EcoRI-pLG_For/09-BamHI-pLG_Rev and trhCBamHiFW/trhCBamHiRV (see S3 Table for the sequences) together with Phusion Hot Start II High-Fidelity DNA Polymerase (Thermo Scientific) following the manufacturer’s recommendations. *rsp* and *trhC* amplification with the above-referred oligonucleotides generated EcoRI/BamHI and BamHI/BamHI sites flanking the *rsp* and *trhC* genes, respectively. The corresponding EcoRI/BamHI and BamHI/BamHI fragments were cloned into the vectors pLG338-30 and pBR322 previously digested with the same enzymes, respectively. The resulting plasmids were Sanger sequenced and termed pLG338-rsp and pBR322-trhC, respectively.

### Polyclonal antibody production

For polyclonal antibody production, the Big 3 domain encoded by the RSP protein was used. The Big 3 domain corresponds to 140 amino acids (Y L Y I F D L T D L T N G S Y A A S F T V E N N S K N T S T Y N E P E S K L M L S D N P T L M V L K D G A A L A K R A P V Y F L N E I I V A A F Q G Q A G V A D I K A V T I D N K L V E L T P T N H K G I Y Y L P V G D D L E V N A D H E I T V I A E N L Y G K I V T F N T T F T Y Q P) and is encoded in the central region of the RSP protein. Amplification of that region was achieved by performing PCR using the R27 plasmid as a DNA template and the primers RSPBig3_31FW and RSPBig3_31RV together with the Thermo Scientific Phusion Hot Start II High-fidelity DNA Polymerase following the manufacturer’s recommendations. The DNA was then purified using a Thermo Scientific GeneJet PCR Purification Kit and ligated into the pLATE31 vector according to the manufacturer´s instructions (Thermo Scientific aLICator LIC cloning and expression system). The resulting plasmid, termed pLATE31-Big3, was Sanger sequenced. BL21 DE3 cells were used for recombinant expression of the Big 3 domain. Cells transformed with pLATE31-Big3 plasmid were grown in LB medium supplemented with carbenicillin at a final concentration of 100 μg/ml at 37°C until O.D._600 nm_ of 0.4. Then, recombinant protein expression was induced by adding IPTG at a final concentration of 1 mM for 3 hours. Cells were then centrifuged at 7,500 xg for 30 minutes at 4°C. The pellet was subsequently resuspended in buffer A20 (20 mM HEPES pH 7.9, 100 mM KCl, 5 mM MgCl_2_, 10% glycerol, 20 mM imidazole) plus protease inhibitor (Complete Ultra Tablets, Mini, EDTA-free, EASYpack, Roche). Cells were then disrupted by sonication, and the insoluble fraction (inclusion bodies) was collected after centrifugation at 12,000 xg for 30 minutes at 4°C. Inclusion bodies containing the recombinant Big 3 protein were solubilized in buffer B (100 mM NaH_2_PO_4_, 10 mM Tris pH 8.0, 8 M urea) for 30 minutes at room temperature. Upon centrifugation (12,000 xg at 4°C for 30 minutes), the supernatant was used for protein purification by by immobilized-metal affinity chromatography (IMAC) using HisPur Ni-NTA Superflow Agarose (Thermo Scientific). Recombinant Big 3 protein was eluted from Ni-NTA resin by changing the pH first using Buffer D (100 mM NaH_2_PO_4_, 10 mM Tris pH 6.3, 8 M urea) and then using buffer E (100 mM NaH_2_PO_4_, 10 mM Tris pH 4.5, 8 M urea). Both eluted fractions were collected and then concentrated using Amicon Ultra-15 Ultracel 3K (Millipore) according to the manufacturer´s instructions. The purified Big 3 protein was adjusted to 1 mg/ml and inoculated into rabbits according to standard protocols (Unitat d'Experimentació Animal de Farmàcia–CCiTUB. Universitat de Barcelona, Barcelona, Spain). After immunization, preimmune serum and serum collected after the immunization period were tested by western blot against the RSP protein.

### Plasmid conjugation

The R27 plasmid was conjugated either in liquid as described previously [45] or on filters in the presence of a physical support (0.45 μm nitrocellulose filters, Millipore). For both protocols, cultures of donor and recipient strains were grown in Penassay broth (1.5 g l^-1^ meat extract, 1.5 g l^-1^ yeast extract, 5 g l^-1^ peptone, 1 g l^-1^ glucose, 3.5 g l^-1^ NaCl, 1.32 g l^-1^ KH_2_PO_4_, 4.82 g l^-1^ K_2_HPO_4_ 3H_2_O). Conjugations were performed using the recipient strains SL1344 ibplac (Km^r^) [38]. Cultures of donor strains SL1344 that harbored the plasmids; R27, R27 Δ*rsp*, R27 Δ*rsp* complemented with pLG338-rsp, R27 Δ*flgE* strain or R27 Δ*rsp* Δ*flgE* strain and recipient strains SL1344 ibplac were grown overnight without shaking at 25°C in Penassay broth. Aliquots were washed to eliminate the antibiotics and resuspended in the same volume of initial culture. In the liquid protocol, 0.4 ml of the recipient strain culture and 0.1 ml of the donor strain culture were mixed and incubated at 25°C without agitation for 2 h. Mixtures were serially diluted and then plated in LB containing either Tc or Tc and Km. The mating frequency was calculated as the number of transconjugants per donor cell. In the filter protocol, 0.4 ml of the recipient strain culture and 0.1 ml of the donor strain culture were mixed. Then, 0.1 ml of the mixture was spotted in the center of a 0.45 μm filter laid on an LB plate. The plates were incubated at 25°C for 16 h. The filters were then washed with 1 ml of 10 mM MgSO_4_, and the cells were collected, serially diluted and plated on LB plates containing either Tc or Tc and Km. The mating frequency was calculated as the number of transconjugants per donor cell. Student´s *t*-test was used to determine statistical significance, and the values were obtained by using the GraphPad Prism 5 software. A *P* value of less than 0.05 was considered significant.

### Oligonucleotides

The oligonucleotides (from 5’ to 3’) used in this work are listed in S3 Table.

### β-Galactosidase assays

β-Galactosidase activity measurements were performed as previously described [63]. Values are given as Miller units. Student´s *t*-test was used to determine statistical significance, and the values were obtained by using the GraphPad Prism 5 software. A *P* value of less than 0.05 was considered significant.

### Motility assay

The motility assay was performed as described [42]. Briefly, motility was performed on tryptone broth (TB) plates (1% tryptone, 0.5% NaCl) containing 0.35% agar. Overnight bacterial cultures grown in LB at 37°C were spotted (5 μl) on the center of the plates and incubated for 24 h at 25°C. The experiments were repeated three times with three plates of each strain in each experiment. The colony diameter was measured and plotted, and standard errors were calculated.

### Flagellum isolation

For flagellum isolation, cells were grown overnight at 25°C in LB medium supplemented with Tc (for R27 selection). Cells were then centrifuged at 8,000 xg for 30 minutes at 4°C. Pellets were resuspended in 1/100 of the initial volume with 100 mM of Tris-HCl pH 8.0 and passed through a 21G syringe six times. Thereafter, the cells were centrifuged (8,000 xg, 20 minutes, 4°C). The resulting supernatants were centrifuged again at 12,000 xg for 30 minutes at 4°C. Again, the supernatants were ultracentrifuged at 40,000 xg for 1 hour at 4°C. The pellet, including the flagella, was resuspended in 100 mM of Tris, 2 mM EDTA pH 8.0 and analyzed by SDS-PAGE with a 10% gel [68].

### Cell-free supernatant (secretome)

Cell-free supernatants were prepared from cultures grown at 25°C until the beginning of stationary phase (O.D._600 nm_ of 2.0). Ten milliliters of bacterial cells were centrifuged, and supernatants were filtered through a 0.22 μm filter (Millipore). For each strain, 2 ml of cell-free supernatants containing secreted proteins were mixed with trichloroacetic acid at a final concentration of 10%. Incubation was performed on ice for 45 minutes, and the tubes were centrifuged for 30 minutes at 12,000 xg at room temperature. The pellets were washed once with cold acetone and again centrifuged for 30 minutes at 12,000 xg at room temperature. Proteins were solubilized with 1x Laemmli Sample Buffer (Bio-Rad). Samples were boiled for 10 minutes and loaded into a SDS-PAGE with a 12.5% gel [68].

### Electrophoresis and western blotting analysis of proteins

Protein samples were analyzed by SDS-PAGE with 10% or 12.5% gels [68]. Proteins were transferred from the gels to PVDF membranes using the Trans-Blot Turbo system (Bio-rad). Western blot analysis was performed with a monoclonal antibody raised against the Flag-epitope (Sigma) diluted 1:10,000 in a solution of PBS, 0.2% Triton, 3% skimmed milk and incubated for 16 hours at 4°C. Membranes were washed for 20 minutes each with PBS, 0.2% Triton solution. The washing step was repeated three times. Thereafter, the membranes were incubated with horseradish peroxidase-conjugated goat anti-mouse IgG (Promega) diluted 1:2500 in a solution of PBS, 0.2% Triton for 1 hour at room temperature. Again, three washing steps of 45 minutes with PBS, 0.2% Triton solution were performed, and detection was performed by enhanced chemiluminescence using ImageQuant LAS54000 imaging system software (GE Healthcare Lifesciences).

### Cell fractionation

Cell fractionation was performed as described [69]. We used 1 ml of bacterial cells from a culture entering stationary phase (O.D._600 nm_ of 2.0) for fractionation. Samples were resolved by SDS-PAGE with a 12.5% gel.

### Protein identification (LC-MS/MS)

Protein identification was performed as described [38].

### Isolation of RNA

Bacterial cells were grown until O.D._600 nm_ of 2.0. Then, 5 ml of cells were then mixed with a 0.2x volume of stop solution buffer (95% ethanol, 5% phenol), shaken and centrifuged (10 minutes, 6,000 x g). Bacterial pellets were subsequently frozen at -80°C until use. Total RNA was extracted from bacterial pellets using Tripure Isolation Reagent (Roche) according to the manufacturer’s instructions. Potential traces of DNA were removed by digestion with DNase I (Turbo DNA-free, Ambion) according to the manufacturer’s instructions. RNA concentration and RNA quality were measured using a Nano-Drop 1000 (Thermo Fisher Scientific).

### Quantitative reverse transcription-PCR (qRT-PCR)

The expression level of the *rsp* gene was determined by using real-time quantitative PCR. Briefly, 1 μg of previously isolated total RNA was reverse transcribed to generate cDNA using a High-capacity cDNA Reverse Transcription kit (Applied Biosystems) according to the manufacturer’s instructions. All samples within an experiment were reverse transcribed at the same time; the resulting cDNA was diluted 1:100 in nuclease-free water and stored in aliquots at –80°C until used. As a control, parallel samples in which reverse transcriptase was omitted from the reaction mixture were run. Real-time PCR was carried out using Maxima SYBR green/ROX qPCR master mix (Thermo Scientific) and an ABI Prism 7700 sequence detection system (Applied Biosystems). Specific oligonucleotides complementary to the genes of interest were designed using primer3 software. Relative quantification of gene expression of mutants versus the wild-type strain was performed using the comparative threshold cycle (CT) method [70]. The relative amount of target cDNA was normalized using the *gapA* gene as an internal reference standard.

### Immunogold electron microscopy

For immunogold labeling of bacterial cells, 10 μl of the different bacterial suspensions were applied to carbon-coated grids for 10 min. Upon removal of the excess of liquid, grids were placed face down on drops of PBS and washed three times (1 minute each). Grids were blocked by floating on drops of PBS containing 1% bovine serum albumin (BSA) for 30 minutes and washed two times in PBS (1 minute each). Grids were then placed on drops of rabbit anti-RSP polyclonal antiserum (1:10 dilution) for 45 min. After three washes in PBS (1 minute each), grids were incubated with goat anti-rabbit IgG conjugated to 12 nm gold particles for 45 min (1:20 dilution). Grids were then washed in PBS and distilled water drops, stained with 2% uranyl acetate and air dried. All incubations were carried out at room temperature. Controls were incubated with goat anti-rabbit IgG conjugated to 12 nm gold particles in the absence of specific antibodies. Samples were examined in a JEOL 1010 transmission electron microscope operating at 80 kV.

### Bioinformatic analysis

The Phyre2 [71] and PSIPRED [72] web portals for protein prediction and analysis were used to predict the secondary structure of the RSP protein. The Conserved Domain algorithm was used to determine putative domains in the RSP sequence (https://www.ncbi.nlm.nih.gov/cdd/). BLASTn was performed using the nucleotide sequence of the *rsp* (*R0009*) gene against the NCBI database, plus setting the search criteria organism to matching only plasmid sequences. To identify the IncHI1 and IncHI2 plasmids among the plasmid database, we used two reference plasmids previously classified by traditional incompatibility typing into the IncHI1 and IncHI2 groups: R27 and R478, respectively. From all plasmids, we retrieved those that met two criteria: (i) they encode proteins that are homologs of more than half of all proteins encoded by any of the reference plasmids, and (ii) they encode replication initiation (Rep) proteins that are homologs to those of the reference plasmids, namely, RepHIA and RepHIB from the IncHI1 plasmids and RepHI2 from the IncHI2 plasmids. Following these parameters, we were able to discriminate those plasmids that share many proteins and encode the same replication machineries as the reference genomes (thus satisfying both criteria) from those that only share many proteins but not the Rep proteins or only Rep but very few other proteins. The results were filtered by a similarity cut-off > 85%, an alignment length between pairs > 85% and an *e*-value < 10^−10^.

### Animals

C57BL/6 mice (both males and females) were obtained from Harlan and maintained under specific pathogen-free conditions at the animal facility of Parc Científic de Barcelona (PCB), Spain.

### Isolation of primary macrophages

Murine bone marrow-derived macrophages (BMDM) were obtained from male and female C57BL/6 mice as previously described [73]. Briefly, bone marrow precursors were differentiated on Petri dishes for 8 days in Dulbecco’s modified Eagle medium (DMEM), supplemented with 20% heat-inactivated fetal bovine serum (FBS) and 30% L-cell-conditioned medium as a source of macrophage-colony-stimulating factor.

### Phagocytosis assay

The *Salmonella* strains SL1344 (R27) and SL1344 (R27 RSP-Flag) were transformed with a plasmid expressing red fluorescent protein (RFP) (pBR-RFP.1) [74] to render bacterial cells fluorescent. Before infection, the strains SL1344 (R27 pBR-RFP.1) and SL1344 (R27 RSP-Flag pBR-RFP.1) were grown at 25°C in the presence of 100 μg/ml of carbenicillin for 16 hours. Bacterial cells were opsonized with anti-Flag antibodies (Sigma-Aldrich) (50 ng/10^6^-CFU) in PBS for 2 hours at 4°C.

Macrophages were plated in 6-well plates (1.5x10^6^ cells/well) containing DMEM-10% FBS for 24 hours before infection, as described [75]. Briefly, 15 minutes before infection, the cells were cooled at 4°C. For infection, either opsonized or nonopsonized bacteria were added to macrophage cultures at a multiplicity of infection of 15. Macrophages were incubated with *Salmonella* cells for 30 min at 37°C and 5% CO_2_. Negative control cells were incubated in the absence of bacteria. Additional control cells were incubated with bacterial cells at 4°C for 30 min.

After infection, noninternalized bacteria were eliminated by three washes with ice cold PBS. Infected cells were fixed in 5% paraformaldehyde. Infection was analyzed by counting cells containing fluorescent bacteria (RFP^+^) in a FacsAria I SORP sorter (Becton Dickinson). Noninfected macrophages were used as a control for autofluorescence. Cells incubated with bacteria at 4°C were used to discriminate bacteria adhered to the macrophage cell surface from internalized bacteria. The infection index was calculated as follows: (%RFP^+^ cells) x (mean fluorescence intensity in the RFP^+^ population). Statistical analysis was performed with GraphPad Prism 7.00. Infection index values were compared using one-way ANOVA and Tukey´s post hoc test for multiple comparisons.

## Supporting information

S1 Fig Phyre2 prediction for the secondary structure of the RSP protein. (DOC)

S2 Fig Similarities of the RSP protein to other proteins as determined by the Phyre2 program. (DOCX)

S3 Fig Physical map of the R27 partitioning/stability (Tra2) region where the *R0009* (*rsp*) gene maps (blue arrow). The figure was not drawn at scale.
(DOCX)

S4 Fig Modification of strain SL1344 motility by the RSP protein. A representative motility experiment showing the different colony diameters after 24 hours of incubation at 25°C is represented. A) Strain SL1344, B) Strain SL1344 (R27), C) Strain SL1344 (R27 Δ*rsp*) and D) Strain SL1344 (R27 Δ*rsp* pLG338-rsp).
(DOCX)

S5 Fig Secreted protein profile of strains SL1344 (R27) (1) and MG1655 (R27) (2). SDS-PAGE analysis of the protein precipitate obtained from of 1 ml of cell-free supernatant. Cells were grown at 25°C until an O.D._600 nm_ of 2.0. Arrow points to the RSP protein.
(DOCX)

S6 Fig SSPred algorithm prediction for RSP protein. Prediction approach was based on the RSP protein amino acid composition. (http://www.bioinformatics.org/sspred/html/sspred.html).
(DOCX)

S7 Fig Flagellum isolation from the strains SL1344, SL1344 (R27), SL1344 (R27 Δ*rsp*) and SL1344 (R27 Δ*rsp*) Δ*flgE*. As indicated by an arrow, RSP co-purifies with flagellins.
(DOCX)

S1 Table BLASTn results corresponding to the alignment of the nucleotide sequence corresponding to the R27 *R0009* gene against the NCBI database. All the indicated plasmids belong to the IncHI group, both IncHI1 (99% identity) and IncHI2 (87% identity). The first 13 entries are shown.
(DOCX)

S2 Table Bacterial strains and plasmids used in this work. (DOCX)

S3 Table Oligonucleotides used in this study. (DOCX)

## References

[1] Morens DM, Folkers GK, Fauci AS. The challenge of emerging and re-emerging infectious diseases. Nature. 2004;430: 242–249. 10.1038/nature02759 15241422 PMC7094993

[2] Meyer E, Schwab F, Schroeren-Boersch B, Gastmeier P. Dramatic increase of third-generation cephalosporin-resistant *E. coli* in German intensive care units: secular trends in antibiotic drug use and bacterial resistance, 2001 to 2008. Crit Care. 2010;14: R113 10.1186/cc9062 20546564 PMC2911759

[3] Rossolini GM, Mantengoli E, Docquier J-D, Musmanno RA, Coratza G. Epidemiology of infections caused by multiresistant gram-negatives: ESBLs, MBLs, panresistant strains. New Microbiol. 2007;30: 332–339. 17802921

[4] Spellberg B, Guidos R, Gilbert D, Bradley J, Boucher HW, Scheld WM, et al The epidemic of antibiotic-resistant infections: a call to action for the medical community from the Infectious Diseases Society of America. Clin Infect Dis. 2008;46: 155–164. 10.1086/524891 18171244

[5] Carattoli A. Plasmids and the spread of resistance. Int J Med Microbiol. 2013;303: 298–304. 10.1016/j.ijmm.2013.02.001 23499304

[6] Wang J, Stephan R, Zurfluh K, Hächler H, Fanning S. Characterization of the genetic environment of bla ESBL genes, integrons and toxin-antitoxin systems identified on large transferrable plasmids in multi-drug resistant *Escherichia coli*. Front Microbiol. 2014;5: 716 10.3389/fmicb.2014.00716 25610429 PMC4285173

[7] Lopatkin AJ, Meredith HR, Srimani JK, Pfeiffer C, Durrett R, You L. Persistence and reversal of plasmid-mediated antibiotic resistance. Nat Commun. 2017;8: 1689 10.1038/s41467-017-01532-1 29162798 PMC5698434

[8] Phan M-D, Wain J. IncHI plasmids, a dynamic link between resistance and pathogenicity. J Infect Dev Ctries. 2008;2: 272–278. 19741288 10.3855/jidc.221

[9] Whiteley M, Taylor DE. Identification of DNA homologies among H incompatibility group plasmids by restriction enzyme digestion and Southern transfer hybridization. Antimicrob Agents Chemother. 1983;24: 194–200. 10.1128/aac.24.2.194 6314885 PMC185137

[10] Liang Q, Yin Z, Zhao Y, Liang L, Feng J, Zhan Z, et al Sequencing and comparative genomics analysis of the IncHI2 plasmids pT5282-mphA and p112298-catA and the IncHI5 plasmid pYNKP001-dfrA. Int J Antimicrob Agents. 2017;49: 709–718. 10.1016/j.ijantimicag.2017.01.021 28390961

[11] Maher D, Sherburne R, Taylor DE. H-pilus assembly kinetics determined by electron microscopy. J Bacteriol. 1993;175: 2175–2183. 10.1128/jb.175.8.2175-2183.1993 8096837 PMC204501

[12] Parry CM, Ho VA, Phuong LT, Bay PVB, Lanh MN, Tung LT, et al Randomized controlled comparison of ofloxacin, azithromycin, and an ofloxacin-azithromycin combination for treatment of multidrug-resistant and nalidixic acid-resistant typhoid fever. Antimicrob Agents Chemother. 2007;51: 819–825. 10.1128/AAC.00447-06 17145784 PMC1803150

[13] Holt KE, Phan M-D, Baker S, Duy PT, Nga TVT, Nair S, et al Emergence of a globally dominant IncHI1 plasmid type associated with multiple drug resistant typhoid. PLoS Negl Trop Dis. 2011;5: e1245 10.1371/journal.pntd.0001245 21811646 PMC3139670

[14] Chen W, Fang T, Zhou X, Zhang D, Shi X, Shi C. IncHI2 Plasmids Are Predominant in Antibiotic-Resistant *Salmonella* Isolates. Front Microbiol. 2016;7: 1566 10.3389/fmicb.2016.01566 27746775 PMC5043248

[15] Villa L, Poirel L, Nordmann P, Carta C, Carattoli A. Complete sequencing of an IncH plasmid carrying the blaNDM-1, blaCTX-M-15 and qnrB1 genes. J Antimicrob Chemother. 2012;67: 1645–1650. 10.1093/jac/dks114 22511638

[16] Dolejska M, Villa L, Poirel L, Nordmann P, Carattoli A. Complete sequencing of an IncHI1 plasmid encoding the carbapenemase NDM-1, the ArmA 16S RNA methylase and a resistance-nodulation-cell division/multidrug efflux pump. J Antimicrob Chemother. 2013;68: 34–39. 10.1093/jac/dks357 22969080

[17] Lim LM, Ly N, Anderson D, Yang JC, Macander L, Jarkowski A, et al Resurgence of colistin: a review of resistance, toxicity, pharmacodynamics, and dosing. Pharmacotherapy. 2010;30: 1279–1291. 10.1592/phco.30.12.1279 21114395 PMC4410713

[18] Catry B, Cavaleri M, Baptiste K, Grave K, Grein K, Holm A, et al Use of colistin-containing products within the European Union and European Economic Area (EU/EEA): development of resistance in animals and possible impact on human and animal health. Int J Antimicrob Agents. 2015;46: 297–306. 10.1016/j.ijantimicag.2015.06.005 26215780

[19] Olaitan AO, Morand S, Rolain J-M. Mechanisms of polymyxin resistance: acquired and intrinsic resistance in bacteria. Front Microbiol. 2014;5: 643 10.3389/fmicb.2014.00643 25505462 PMC4244539

[20] Liu Y-Y, Wang Y, Walsh TR, Yi L-X, Zhang R, Spencer J, et al Emergence of plasmid-mediated colistin resistance mechanism MCR-1 in animals and human beings in China: a microbiological and molecular biological study. Lancet Infect Dis. 2016;16: 161–168. 10.1016/S1473-3099(15)00424-7 26603172

[21] Gao R, Hu Y, Li Z, Sun J, Wang Q, Lin J, et al Dissemination and Mechanism for the MCR-1 Colistin Resistance. PLoS Pathog. 2016;12: e1005957 10.1371/journal.ppat.1005957 27893854 PMC5125707

[22] Xavier BB, Lammens C, Ruhal R, Kumar-Singh S, Butaye P, Goossens H, et al Identification of a novel plasmid-mediated colistin-resistance gene, *mcr-2*, in *Escherichia coli*, Belgium, June 2016. Euro Surveill. 2016;21 10.2807/1560-7917.ES.2016.21.27.30280 27416987

[23] Borowiak M, Fischer J, Hammerl JA, Hendriksen RS, Szabo I, Malorny B. Identification of a novel transposon-associated phosphoethanolamine transferase gene, *mcr-5*, conferring colistin resistance in d-tartrate fermenting *Salmonella enterica* subsp. enterica serovar Paratyphi B. J Antimicrob Chemother. 2017;72: 3317–3324. 10.1093/jac/dkx327 28962028

[24] Carattoli A, Villa L, Feudi C, Curcio L, Orsini S, Luppi A, et al Novel plasmid-mediated colistin resistance *mcr-4* gene in *Salmonella* and *Escherichia coli*, Italy 2013, Spain and Belgium, 2015 to 2016. Euro Surveill. 2017;22 10.2807/1560-7917.ES.2017.22.31.30589 28797329 PMC5553062

[25] Yin W, Li H, Shen Y, Liu Z, Wang S, Shen Z, et al Novel Plasmid-Mediated Colistin Resistance Gene *mcr-3* in *Escherichia coli*. MBio. 2017;8 10.1128/mBio.00543-17 28655818 PMC5487729

[26] Matamoros S, van Hattem JM, Arcilla MS, Willemse N, Melles DC, Penders J, et al Global phylogenetic analysis of *Escherichia coli* and plasmids carrying the *mcr-1* gene indicates bacterial diversity but plasmid restriction. Sci Rep. 2017;7: 15364 10.1038/s41598-017-15539-7 29127343 PMC5681592

[27] Wong MH-Y, Chan EW-C, Xie L, Li R, Chen S. IncHI2 Plasmids Are the Key Vectors Responsible for *oqxAB* Transmission among *Salmonella* Species. Antimicrob Agents Chemother. 2016;60: 6911–6915. 10.1128/AAC.01555-16 27572409 PMC5075106

[28] Wang Y, Tian G-B, Zhang R, Shen Y, Tyrrell JM, Huang X, et al Prevalence, risk factors, outcomes, and molecular epidemiology of mcr-1-positive Enterobacteriaceae in patients and healthy adults from China: an epidemiological and clinical study. Lancet Infect Dis. 2017;17: 390–399. 10.1016/S1473-3099(16)30527-8 28139431

[29] Zheng B, Dong H, Xu H, Lv J, Zhang J, Jiang X, et al Coexistence of MCR-1 and NDM-1 in Clinical *Escherichia coli* Isolates. Clin Infect Dis. 2016;63: 1393–1395. 10.1093/cid/ciw553 27506685

[30] Forde BM, Zowawi HM, Harris PNA, Roberts L, Ibrahim E, Shaikh N, et al Discovery of *mcr-1*-Mediated Colistin Resistance in a Highly Virulent *Escherichia coli* Lineage. mSphere. 2018;3 10.1128/mSphere.00486-18 30305321 PMC6180223

[31] Lawley TD, Gilmour MW, Gunton JE, Standeven LJ, Taylor DE. Functional and mutational analysis of conjugative transfer region 1 (Tra1) from the IncHI1 plasmid R27. J Bacteriol. 2002;184: 2173–2180. 10.1128/JB.184.8.2173-2180.2002 11914349 PMC134963

[32] Lawley TD, Gilmour MW, Gunton JE, Tracz DM, Taylor DE. Functional and mutational analysis of conjugative transfer region 2 (Tra2) from the IncHI1 plasmid R27. J Bacteriol. 2003;185: 581–591. 10.1128/JB.185.2.581-591.2003 12511505 PMC145343

[33] Alonso G, Baptista K, Ngo T, Taylor DE. Transcriptional organization of the temperature-sensitive transfer system from the IncHI1 plasmid R27. Microbiology (Reading, Engl). 2005;151: 3563–3573. 10.1099/mic.0.28256-0 16272379

[34] Sherburne CK, Lawley TD, Gilmour MW, Blattner FR, Burland V, Grotbeck E, et al The complete DNA sequence and analysis of R27, a large IncHI plasmid from *Salmonella* typhi that is temperature sensitive for transfer. Nucleic Acids Res. 2000;28: 2177–2186. 10.1093/nar/28.10.2177 10773089 PMC105367

[35] Gilmour MW, Thomson NR, Sanders M, Parkhill J, Taylor DE. The complete nucleotide sequence of the resistance plasmid R478: defining the backbone components of incompatibility group H conjugative plasmids through comparative genomics. Plasmid. 2004;52: 182–202. 10.1016/j.plasmid.2004.06.006 15518875

[36] Halaby DM, Mornon JP. The immunoglobulin superfamily: an insight on its tissular, species, and functional diversity. J Mol Evol. 1998;46: 389–400. 10.1007/pl00006318 9541533

[37] Bodelón G, Palomino C, Fernández LÁ. Immunoglobulin domains in *Escherichia coli* and other enterobacteria: from pathogenesis to applications in antibody technologies. FEMS Microbiol Rev. 2013;37: 204–250. 10.1111/j.1574-6976.2012.00347.x 22724448

[38] Hüttener M, Prieto A, Aznar S, Dietrich M, Paytubi S, Juárez A. Tetracycline alters gene expression in *Salmonella* strains that harbor the Tn10 transposon. Environ Microbiol Rep. 2018;10: 202–209. 10.1111/1758-2229.12621 29393572

[39] Yang Y-H, Jiang Y-L, Zhang J, Wang L, Bai X-H, Zhang S-J, et al Structural insights into SraP-mediated *Staphylococcus aureus* adhesion to host cells. PLoS Pathog. 2014;10: e1004169 10.1371/journal.ppat.1004169 24901708 PMC4047093

[40] Wagner C, Polke M, Gerlach RG, Linke D, Stierhof Y-D, Schwarz H, et al Functional dissection of SiiE, a giant non-fimbrial adhesin of *Salmonella enterica*. Cell Microbiol. 2011;13: 1286–1301. 10.1111/j.1462-5822.2011.01621.x 21729227

[41] Rooker MM, Sherburne C, Lawley TD, Taylor DE. Characterization of the Tra2 region of the IncHI1 plasmid R27. Plasmid. 1999;41: 226–239. 10.1006/plas.1999.1396 10366528

[42] Paytubi S, Aznar S, Madrid C, Balsalobre C, Dillon SC, Dorman CJ, et al A novel role for antibiotic resistance plasmids in facilitating *Salmonella* adaptation to non-host environments. Environ Microbiol. 2014;16: 950–962. 10.1111/1462-2920.12244 24024872

[43] Taylor DE, Newnham PJ, Sherburne C, Lawley TD, Rooker MM. Sequencing and characterization of *Salmonella* typhi plasmid R27 (incompatibility group HI1) *trhC*, a transfer gene encoding a potential nucleoside triphosphate-binding domain. Plasmid. 1999;41: 207–218. 10.1006/plas.1999.1394 10366526

[44] Forns N, Baños RC, Balsalobre C, Juárez A, Madrid C. Temperature-dependent conjugative transfer of R27: role of chromosome- and plasmid-encoded Hha and H-NS proteins. J Bacteriol. 2005;187: 3950–3959. 10.1128/JB.187.12.3950-3959.2005 15937157 PMC1151748

[45] Taylor DE, Levine JG. Studies of temperature-sensitive transfer and maintenance of H incompatibility group plasmids. J Gen Microbiol. 1980;116: 475–484. 10.1099/00221287-116-2-475 6989956

[46] Maher D, Taylor DE. Host range and transfer efficiency of incompatibility group HI plasmids. Can J Microbiol. 1993;39: 581–587. 10.1139/m93-084 8358670

[47] Vogel JP, Andrews HL, Wong SK, Isberg RR. Conjugative transfer by the virulence system of *Legionella pneumophila*. Science. 1998;279: 873–876. 10.1126/science.279.5352.873 9452389

[48] Hong PC, Tsolis RM, Ficht TA. Identification of genes required for chronic persistence of *Brucella abortus* in mice. Infect Immun. 2000;68: 4102–4107. 10.1128/iai.68.7.4102-4107.2000 10858227 PMC101704

[49] Fernández-González E, de Paz HD, Alperi A, Agúndez L, Faustmann M, Sangari FJ, et al Transfer of R388 derivatives by a pathogenesis-associated type IV secretion system into both bacteria and human cells. J Bacteriol. 2011;193: 6257–6265. 10.1128/JB.05905-11 21908662 PMC3209219

[50] Schröder G, Schuelein R, Quebatte M, Dehio C. Conjugative DNA transfer into human cells by the VirB/VirD4 type IV secretion system of the bacterial pathogen *Bartonella henselae*. Proceedings of the National Academy of Sciences. 2011;108: 14643–14648. 10.1073/pnas.1019074108 21844337 PMC3167556

[51] Christie PJ. The Mosaic Type IV Secretion Systems. EcoSal Plus. 2016;7 10.1128/ecosalplus.ESP-0020-2015 27735785 PMC5119655

[52] Cascales E, Christie PJ. The versatile bacterial type IV secretion systems. Nat Rev Microbiol. 2003;1: 137–149. 10.1038/nrmicro753 15035043 PMC3873781

[53] Luque A, Paytubi S, Sánchez-Montejo J, Gibert M, Balsalobre C, Madrid C. Crosstalk between bacterial conjugation and motility is mediated by plasmid-borne regulators. Environ Microbiol Rep. 2019 10.1111/1758-2229.12784 31309702

[54] Siboo IR, Chambers HF, Sullam PM. Role of SraP, a Serine-Rich Surface Protein of *Staphylococcus aureus*, in binding to human platelets. Infect Immun. 2005;73: 2273–2280. 10.1128/IAI.73.4.2273-2280.2005 15784571 PMC1087419

[55] Li X, Bleumink-Pluym NMC, Luijkx YMCA, Wubbolts RW, van Putten JPM, Strijbis K. MUC1 is a receptor for the *Salmonella* SiiE adhesin that enables apical invasion into enterocytes. PLoS Pathog. 2019;15: e1007566 10.1371/journal.ppat.1007566 30716138 PMC6375660

[56] Walsh PM, McKay LL. Recombinant plasmid associated cell aggregation and high-frequency conjugation of *Streptococcus lactis* ML3. J Bacteriol. 1981;146: 937–944. 6787018 10.1128/jb.146.3.937-944.1981 PMC216947

[57] Luo H, Wan K, Wang HH. High-frequency conjugation system facilitates biofilm formation and pAMbeta1 transmission by *Lactococcus lactis*. Appl Environ Microbiol. 2005;71: 2970–2978. 10.1128/AEM.71.6.2970-2978.2005 15932992 PMC1151824

[58] Tremblay C-L, Archambault M. Interference in pheromone-responsive conjugation of a high-level bacitracin resistant *Enterococcus faecalis* plasmid of poultry origin. Int J Environ Res Public Health. 2013;10: 4245–4260. 10.3390/ijerph10094245 24030654 PMC3799527

[59] Tietgen M, Semmler T, Riedel-Christ S, Kempf VAJ, Molinaro A, Ewers C, et al Impact of the colistin resistance gene *mcr-1* on bacterial fitness. Int J Antimicrob Agents. 2018;51: 554–561. 10.1016/j.ijantimicag.2017.11.011 29180279

[60] Arcilla MS, van Hattem JM, Matamoros S, Melles DC, Penders J, de Jong MD, et al Dissemination of the *mcr-1* colistin resistance gene. Lancet Infect Dis. 2016;16: 147–149. 10.1016/S1473-3099(15)00541-1 26711361

[61] Baquero F, Coque TM, la Cruz de F. Ecology and evolution as targets: the need for novel eco-evo drugs and strategies to fight antibiotic resistance. Antimicrob Agents Chemother. 2011;55: 3649–3660. 10.1128/AAC.00013-11 21576439 PMC3147629

[62] Lipsitch M, Siber GR. How Can Vaccines Contribute to Solving the Antimicrobial Resistance Problem? MBio. 2016;7 10.1128/mBio.00428-16 27273824 PMC4959668

[63] Miller JH. A Short Course in Bacterial Genetics. CSHL Press; 1992.

[64] Datsenko KA, Wanner BL. One-step inactivation of chromosomal genes in *Escherichia coli* K-12 using PCR products. Proc Natl Acad Sci USA. 2000;97: 6640–6645. 10.1073/pnas.120163297 10829079 PMC18686

[65] Cherepanov PP, Wackernagel W. Gene disruption in *Escherichia coli*: Tc^R^ and Km^R^ cassettes with the option of Flp-catalyzed excision of the antibiotic-resistance determinant. Gene. 1995;158: 9–14. 10.1016/0378-1119(95)00193-a 7789817

[66] Ellermeier CD, Janakiraman A, Slauch JM. Construction of targeted single copy lac fusions using lambda Red and FLP-mediated site-specific recombination in bacteria. Gene. 2002;290: 153–161. 10.1016/s0378-1119(02)00551-6 12062810

[67] Uzzau S, Figueroa-Bossi N, Rubino S, Bossi L. Epitope tagging of chromosomal genes in *Salmonella*. Proc Natl Acad Sci USA. 2001;98: 15264–15269. 10.1073/pnas.261348198 11742086 PMC65018

[68] Sambrook J, Russell DW. Molecular Cloning. CSHL Press; 2001 10.1089/152045501300189286

[69] Wai SN, Westermark M, Oscarsson J, Jass J, Maier E, Benz R, et al Characterization of dominantly negative mutant ClyA cytotoxin proteins in *Escherichia coli*. J Bacteriol. 2003;185: 5491–5499. 10.1128/JB.185.18.5491-5499.2003 12949101 PMC193753

[70] Livak KJ, Schmittgen TD. Analysis of relative gene expression data using real-time quantitative PCR and the 2(-Delta Delta C(T)) Method. Methods. 2001;25: 402–408. 10.1006/meth.2001.1262 11846609

[71] Kelley LA, Mezulis S, Yates CM, Wass MN, Sternberg MJE. The Phyre2 web portal for protein modeling, prediction and analysis. Nat Protoc. 2015;10: 845–858. 10.1038/nprot.2015.053 25950237 PMC5298202

[72] Jones DT. Protein secondary structure prediction based on position-specific scoring matrices. J Mol Biol. 1999;292: 195–202. 10.1006/jmbi.1999.3091 10493868

[73] Valledor AF, Comalada M, Xaus J, Celada A. The differential time-course of extracellular-regulated kinase activity correlates with the macrophage response toward proliferation or activation. J Biol Chem. 2000;275: 7403–7409. 10.1074/jbc.275.10.7403 10702314

[74] Birmingham CL, Smith AC, Bakowski MA, Yoshimori T, Brumell JH. Autophagy controls *Salmonella* infection in response to damage to the *Salmonella*-containing vacuole. J Biol Chem. 2006;281: 11374–11383. 10.1074/jbc.M509157200 16495224

[75] Matalonga J, Glaria E, Bresque M, Escande C, Carbó JM, Kiefer K, et al The Nuclear Receptor LXR Limits Bacterial Infection of Host Macrophages through a Mechanism that Impacts Cellular NAD Metabolism. Cell Rep. 2017;18: 1241–1255. 10.1016/j.celrep.2017.01.007 28147278

