# Supplementary figures and images for: Expression of a novel class of bacterial Ig-like proteins is required for IncHI plasmid conjugation
Source: PLoS Genet. 2019 Sep 17;15(9):e1008399. doi: 10.1371/journal.pgen.1008399 (PMC6764697; doi:10.1371/journal.pgen.1008399)

**S1A Figure.**


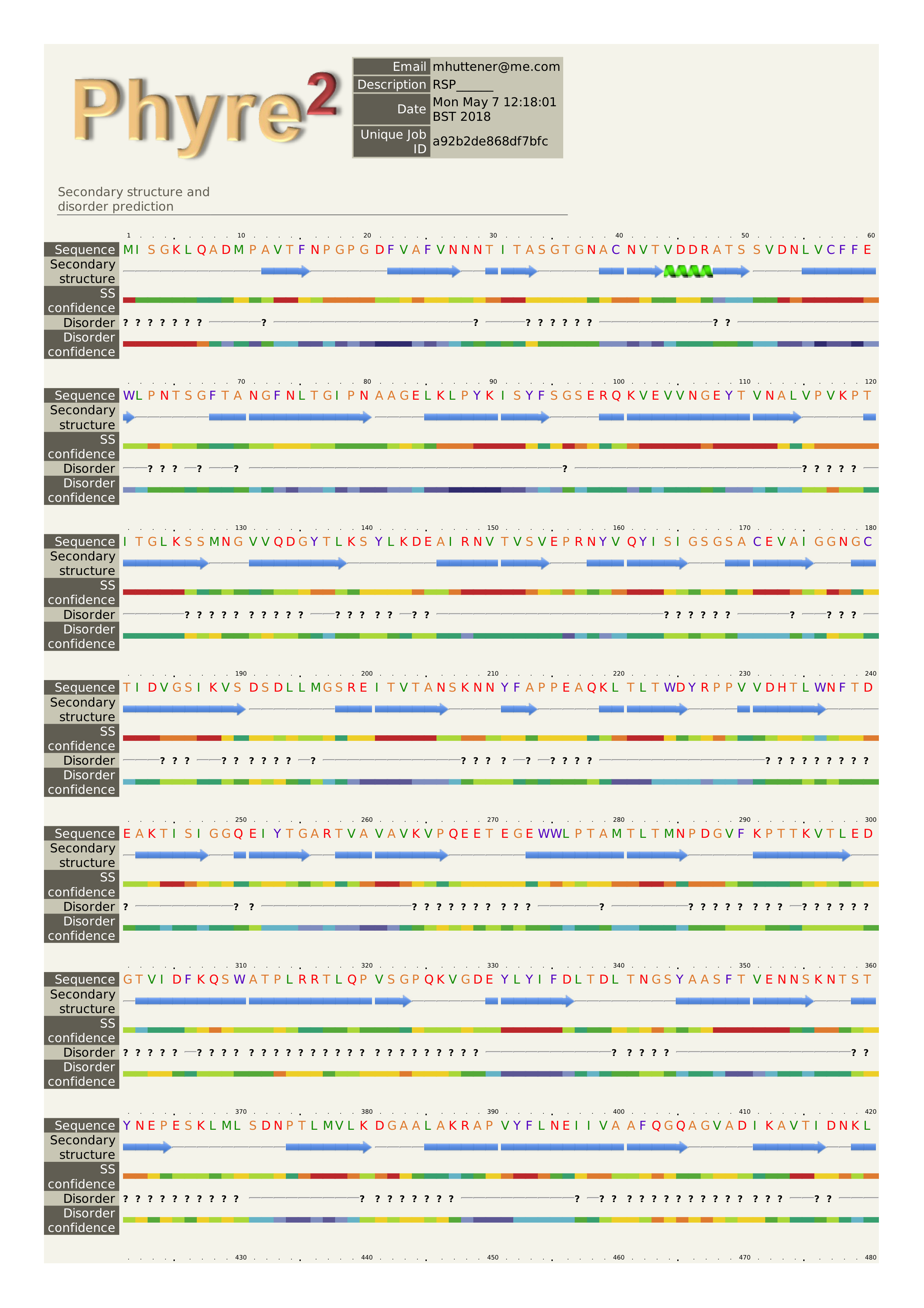


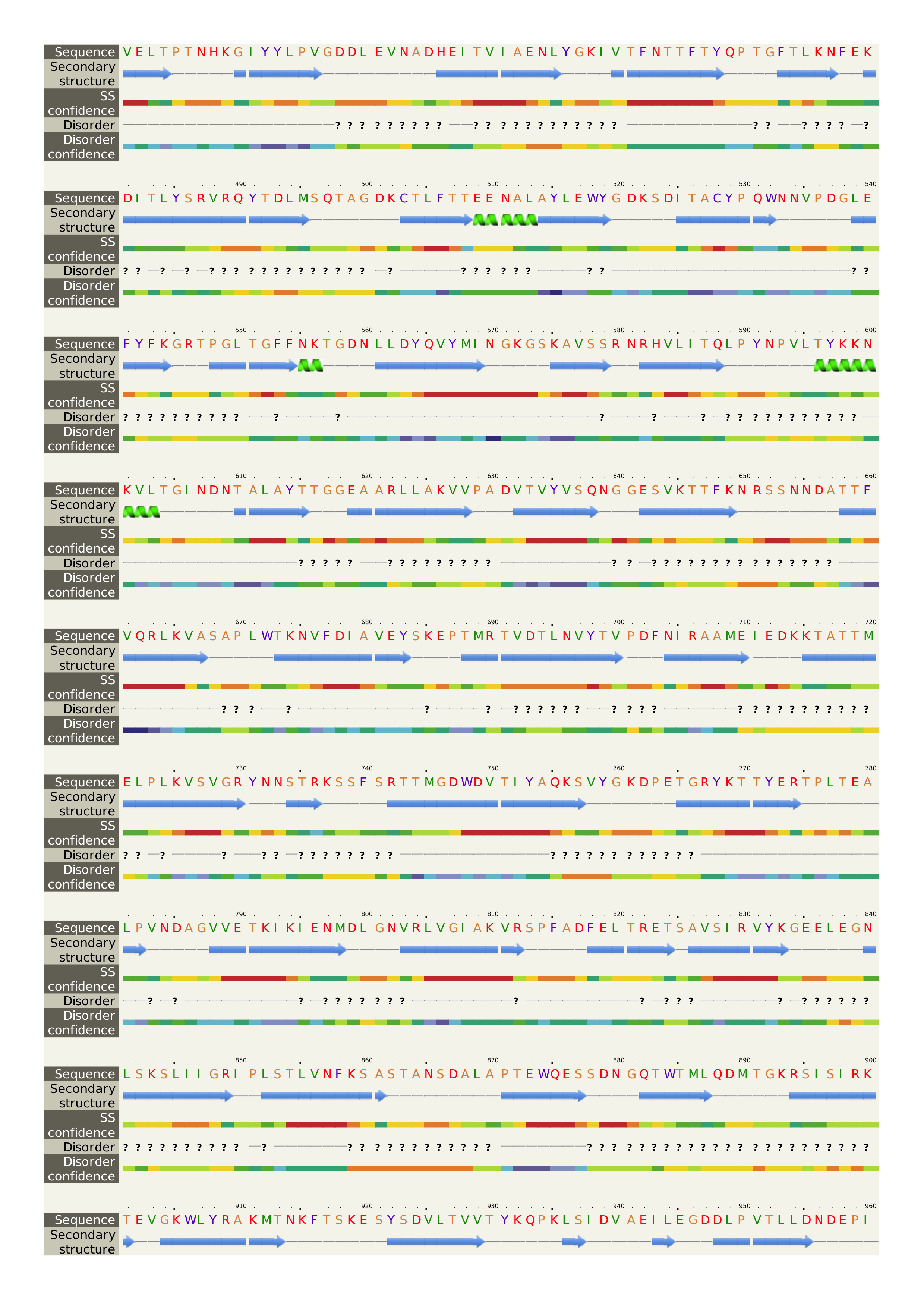


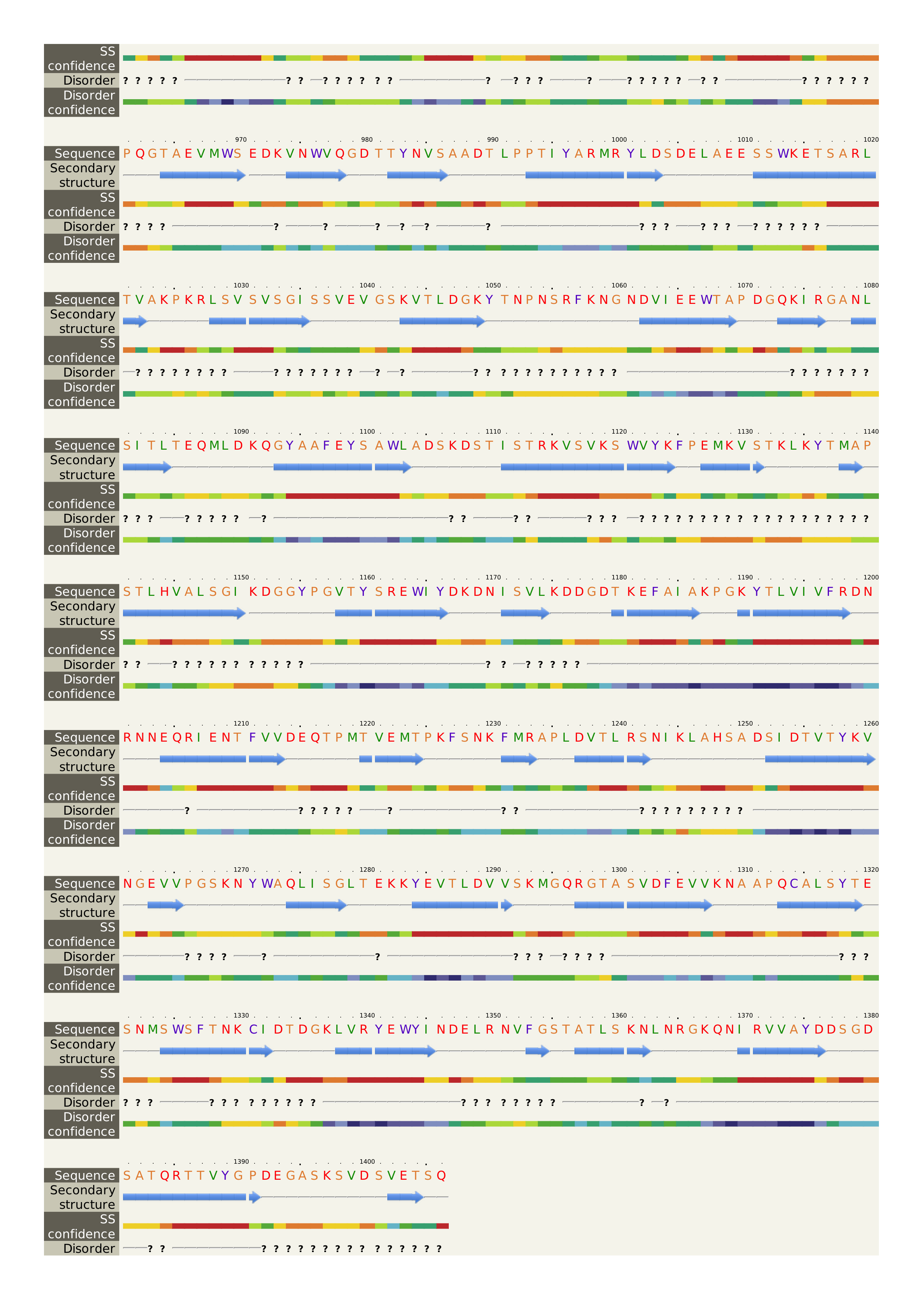


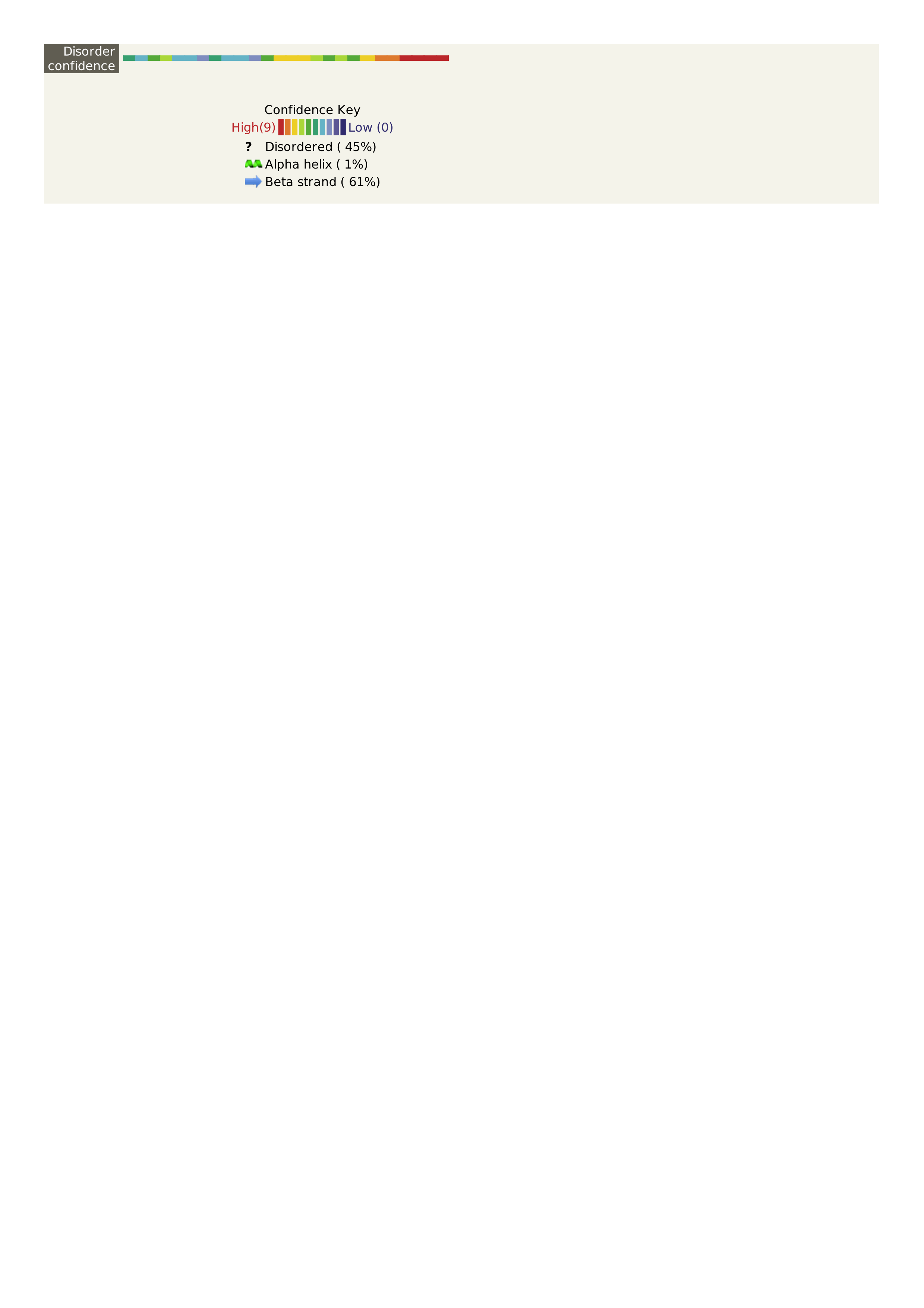

Supplement: S1 Fig — (DOC) [file pgen.1008399.s001.doc]

**S1B Figure.**


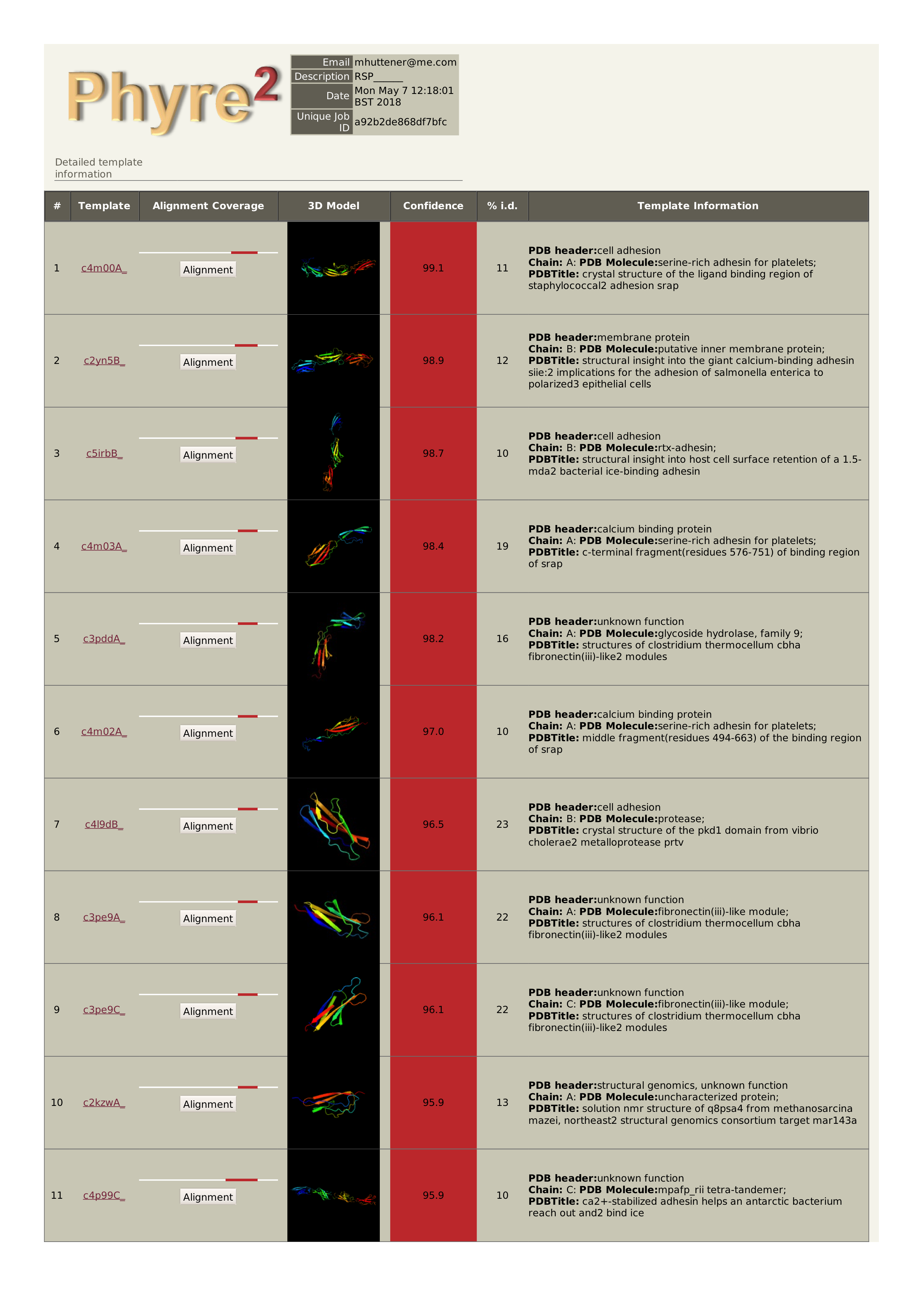


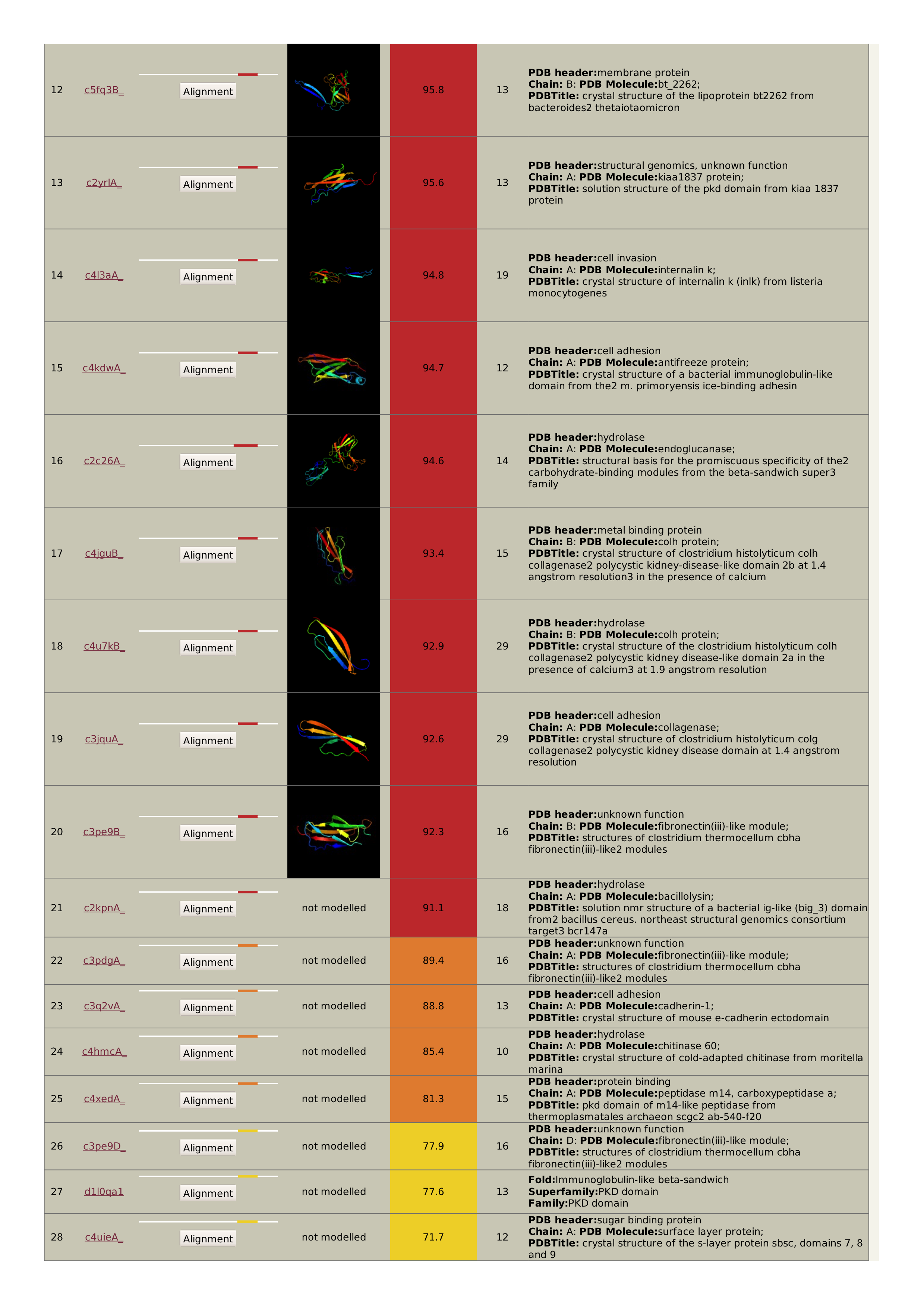


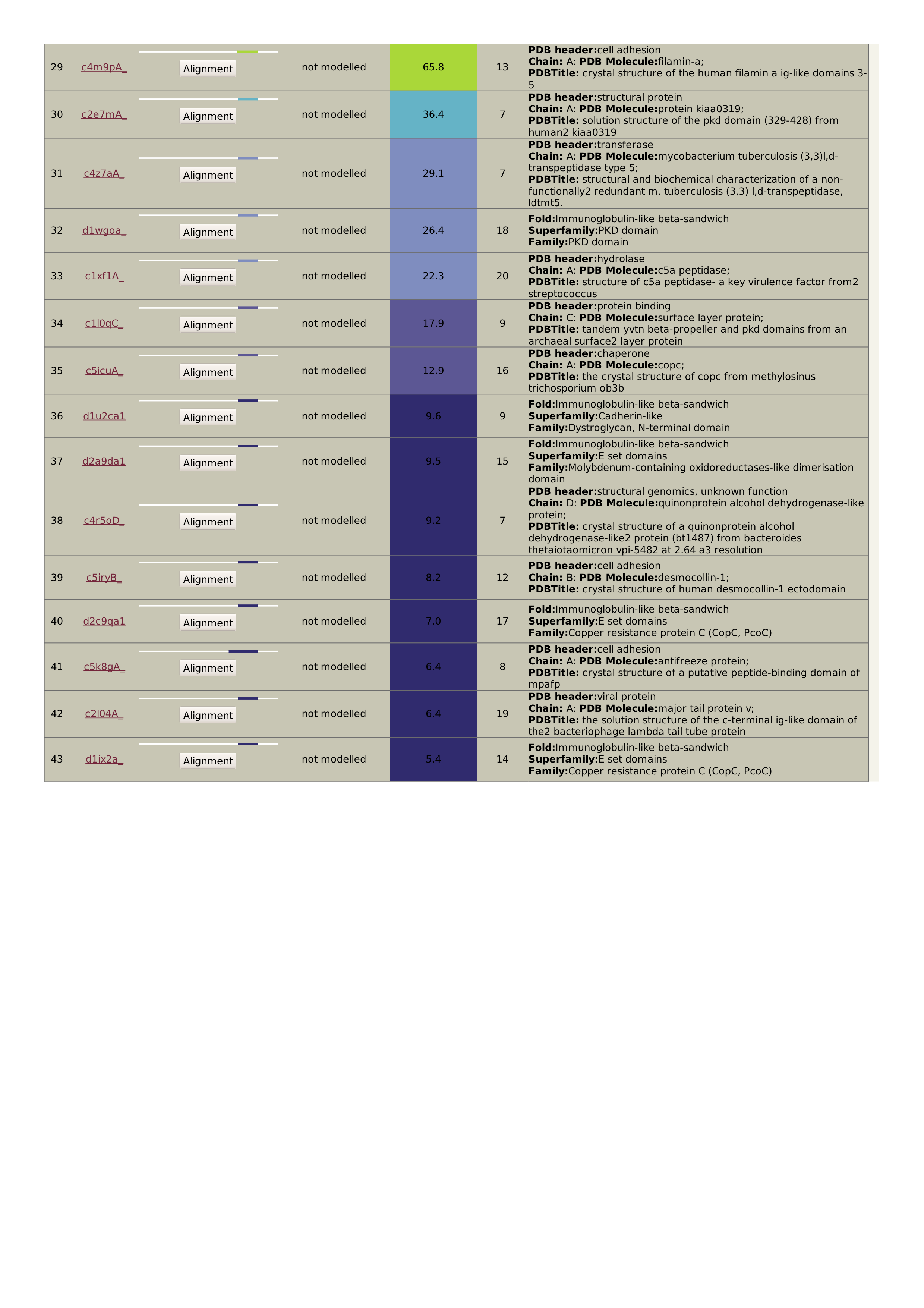

Supplement: S2 Fig — (DOCX) [file pgen.1008399.s002.docx]

**S1C Figure.**


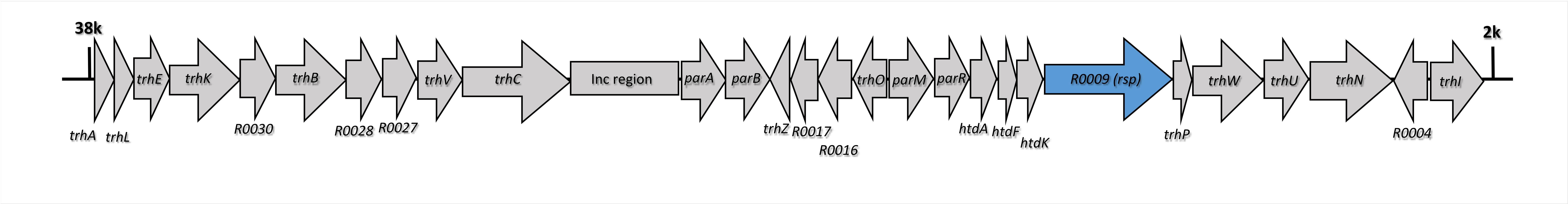

Supplement: S3 Fig — The figure was not drawn at scale. (DOCX) [file pgen.1008399.s003.docx]

**S1D Figure.**


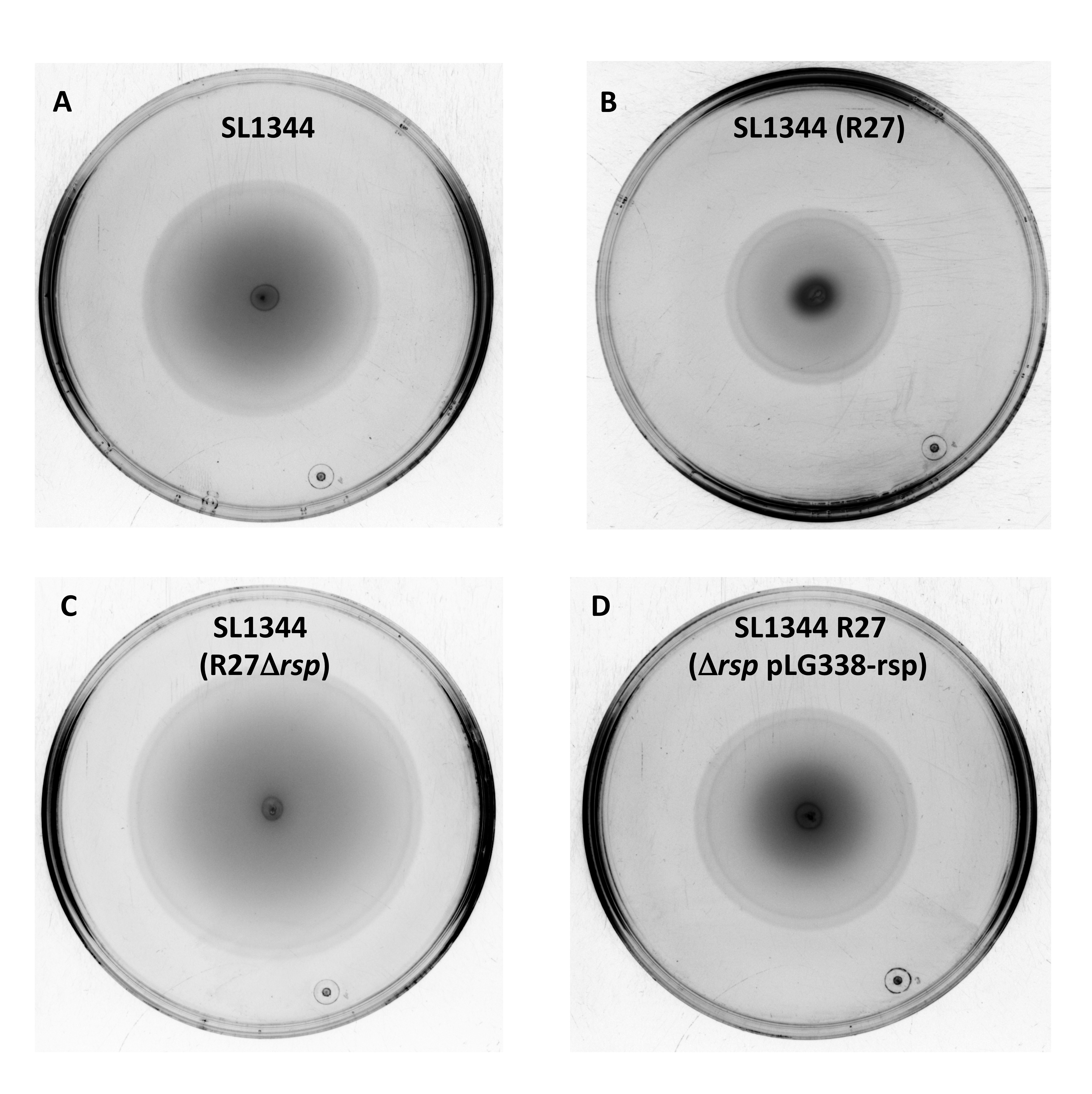

Supplement: S4 Fig — A representative motility experiment showing the different colony diameters after 24 hours of incubation at 25°C is represented. A) Strain SL1344, B) Strain SL1344 (R27), C) Strain SL1344 (R27 Δrsp) and D) Strain SL1344 (R27 Δrsp pLG338-rsp). (DOCX) [file pgen.1008399.s004.docx]

**S1E Figure.**


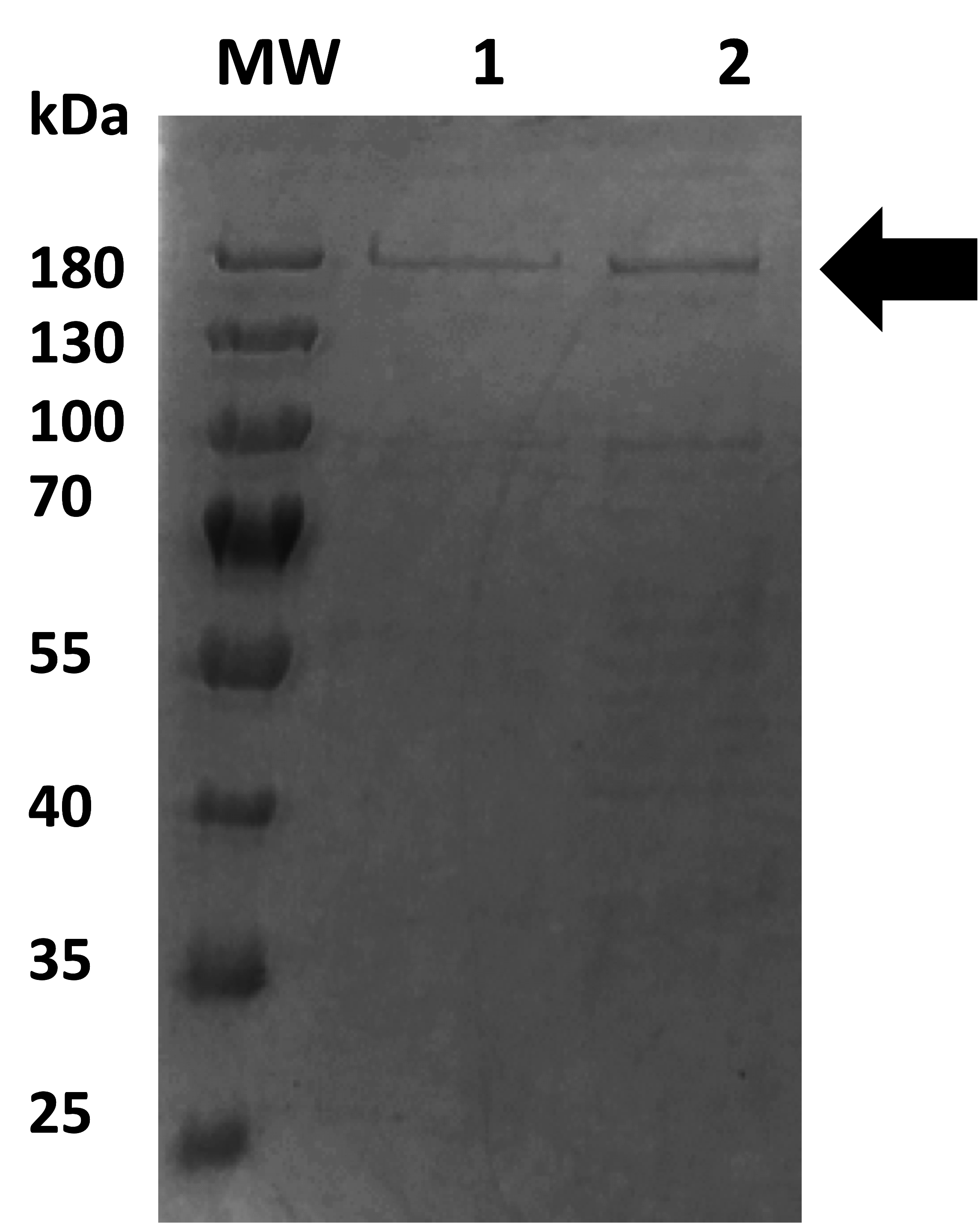

Supplement: S5 Fig — SDS-PAGE analysis of the protein precipitate obtained from of 1 ml of cell-free supernatant. Cells were grown at 25°C until an O.D.600 nm of 2.0. Arrow points to the RSP protein. (DOCX) [file pgen.1008399.s005.docx]

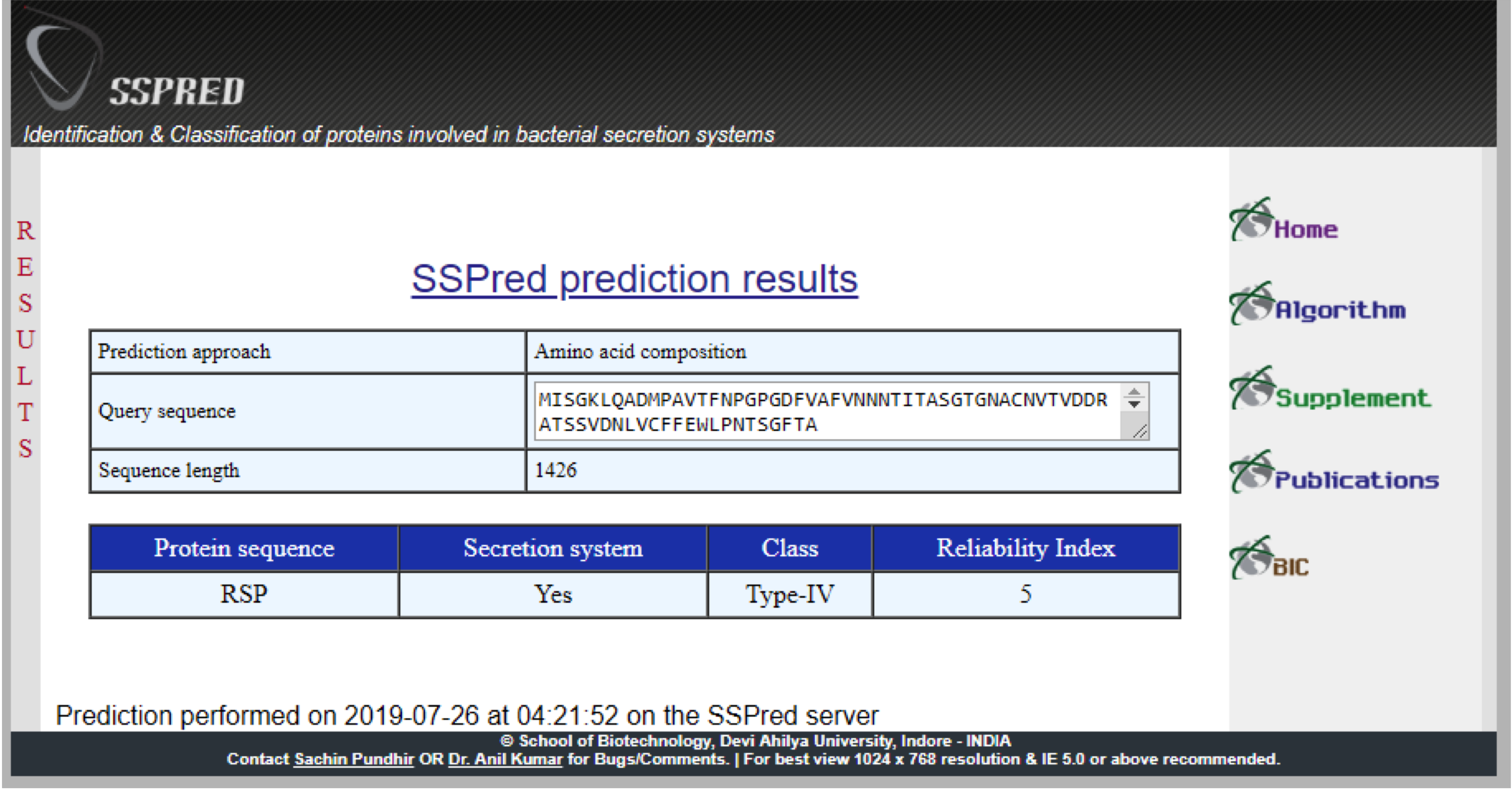
**S1F Figure.**

Supplement: S6 Fig — Prediction approach was based on the RSP protein amino acid composition. (http://www.bioinformatics.org/sspred/html/sspred.html). (DOCX) [file pgen.1008399.s006.docx]

**S1G Figure.**


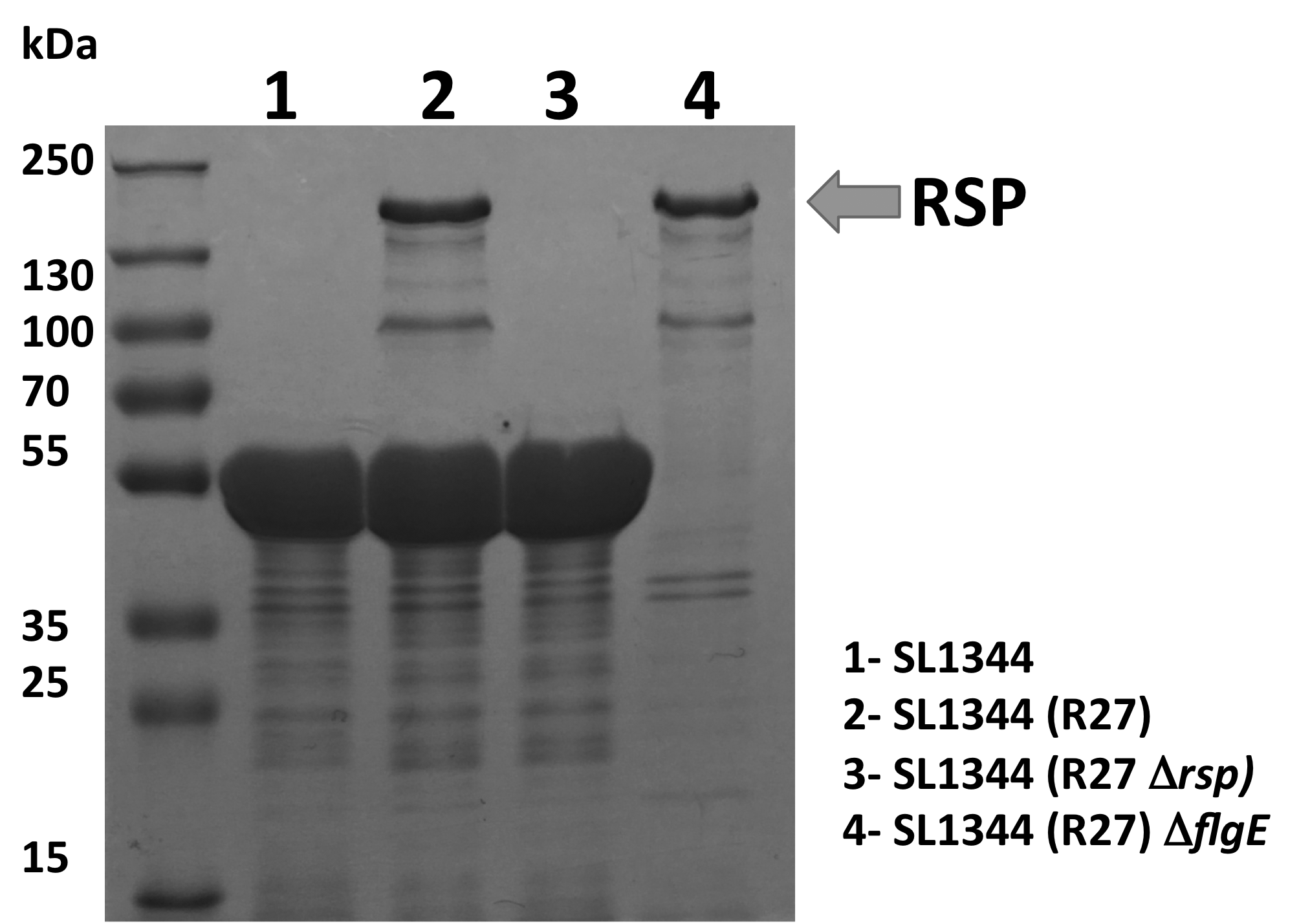

Supplement: S7 Fig — As indicated by an arrow, RSP co-purifies with flagellins. (DOCX) [file pgen.1008399.s007.docx]
