# Supplementary material for: Expression of a novel class of bacterial Ig-like proteins is required for IncHI plasmid conjugation
Source: PLoS Genet. 2019 Sep 17;15(9):e1008399. doi: 10.1371/journal.pgen.1008399 (PMC6764697; doi:10.1371/journal.pgen.1008399)
Supplement: S1 Table — All the indicated plasmids belong to the IncHI group, both IncHI1 (99% identity) and IncHI2 (87% identity). The first 13 entries are shown. (DOCX) [file pgen.1008399.s008.docx]

**S1 Table.**

| **Description** | **Max score** | **Total score** | **Query cover** | **E value** | **Ident.** | **Accession** |
| --- | --- | --- | --- | --- | --- | --- |
| *Salmonella* Typhi R27 plasmid | 7613 | 7613 | 100% | 0.0 | 100% | AF250878.1 |
| *E. coli* H226B plasmid pH226B | 7598 | 7598 | 100% | 0.0 | 99% | KX129784.1 |
| *Salmonella* enterica B71 plasmid pB71 | 7598 | 7598 | 100% | 0.0 | 99% | KP899806.1 |
| *Salmonella* enterica 109/9 plasmid 109/9 | 7598 | 7598 | 100% | 0.0 | 99% | KP899805.1 |
| *Salmonella* enterica F8475 plasmid pF8475 | 7598 | 7598 | 100% | 0.0 | 99% | KP899804.1 |
| *E. coli* 63743 plasmid pEQ2 | 7598 | 7598 | 100% | 0.0 | 99% | KF362122.2 |
| *E. coli* T23 plasmid pEQ1 | 7598 | 7598 | 100% | 0.0 | 99% | KF362121.2 |
| *Salmonella* enterica L-2454 plasmid pMAK1 | 7598 | 7598 | 100% | 0.0 | 99% | AB366440.1 |
| *E. coli* EC2 plasmid pEC2 | 7595 | 7595 | 100% | 0.0 | 99% | CP016183.1 |
| *Salmonella* Typhi P-stx-12 plasmid | 7589 | 7589 | 100% | 0.0 | 99% | CP003279.1 |
| *Salmonella* Typhi CT18 pHCM1 plasmid | 7586 | 7586 | 100% | 0.0 | 99% | AL513383.1 |
| *Salmonella* enterica 8025 plasmid p8025 | 5160 | 5160 | 100% | 0.0 | 87% | KP899803.1 |
| *Citrobacter freundii* plasmid pNDM-CIT | 5160 | 5160 | 100% | 0.0 | 87% | JX182975.1 |
