# Supplementary material for: Expression of a novel class of bacterial Ig-like proteins is required for IncHI plasmid conjugation
Source: PLoS Genet. 2019 Sep 17;15(9):e1008399. doi: 10.1371/journal.pgen.1008399 (PMC6764697; doi:10.1371/journal.pgen.1008399)
Supplement: S2 Table — (DOCX) [file pgen.1008399.s009.docx]

**S2 Table.**

| **Strains** | **Relevant characteristics** | **Reference/Source** |
| --- | --- | --- |
| SL1344 | *rspLhisG* | [1] |
| SL1344 ibplac | *ibpA_420_::lacZ.* Km^r^ | [2] |
| MG1655 | *F-, ilvG, rph1* | [3] |
| SL1344 Δ*flgE* | *flgE::*FRT | This work |
| BL21 DE3 | *hsdS, gal, (λclts857, ind1, Sam7, nin5, lac-UV5-T7gene1)* | [4] |
| **Plasmids** | **Relevant characteristics** | **Reference/Source** |
| R27 | IncHI1, Tc^r^ | [5] |
| R27 rsp | R27 *rsp*::Cm, Cm^r^, Tc^r^ | This work |
| R27 Δ*rsp* | R27 *rsp*::FRT, Tc^r^ | This work |
| R27 rsplacZ | R27 *rsp::lacZ.* Km^r^, Tc^r^ | This work |
| R27 Δ*trhC* | R27 *trhC*::FRT, Tc^r^ | This work |
| R27 RSP-Flag | R27 *rsp*::Flag, Km^r^, Tc^r^ | This work |
| R27 trhC-RSP | *trhC*::Cm, *rsp*::Flag. Cm^r^, Km^r^, Tc^r^ | This work |
| pLG338-30 | oripSC101. Cbr | [6] |
| pLG338-rsp | pLG338-30 + *rsp* from R27 | This work |
| pBR322 | ori_p_MB1, Tc^r^, Cb^r^ | [7] |
| pBR322-trhC | pBR322 + *trhC* from R27 | This work |
| pKD4 | *bla* FRT *ahp* FRT PS1 PS2 oriR6K Km^r^, Cb^r^ |  |
| pKD3 | *bla* FRT *cat* FRT PS1 PS2 oriR6K Cm^r^, Cb^r^ | [8] |
| pSUB11 | Flag- and Km^r^-coding template vector | [9] |
| pKD46 | *oriR101, repA101 (ts), AraBp-gam-bet-exo* | [8] |
| pKG136 | template for transcriptional *lacZ* fusion | [10] |
| pBR-RFP.1 | pBR322, *rpsM* promoter, Cb^r^ | [11] |

**References**

1. Hoiseth SK, Stocker BA. Aromatic-dependent *Salmonella* typhimurium are non-virulent and effective as live vaccines. Nature. 1981;291: 238–239.

2. Hüttener M, Prieto A, Aznar S, Dietrich M, Paytubi S, Juárez A. Tetracycline alters gene expression in *Salmonella* strains that harbor the Tn*10* transposon. Environ Microbiol Rep. 2018;10: 202–209. doi:10.1111/1758-2229.12621

3. Guyer MS, Reed RR, Steitz JA, Low KB. Identification of a sex-factor-affinity site in *E. coli* as gamma delta. Cold Spring Harb Symp Quant Biol. 1981;45 Pt 1: 135–140.

4. Studier FW, Moffatt BA. Use of bacteriophage T7 RNA polymerase to direct selective high-level expression of cloned genes. J Mol Biol. 1986;189: 113–130.

5. Grindley N, Grindley JN, Anderson ES. R factor compatibility groups. Molecular and General Genet. 1972;119: 287–297. doi:10.1007/BF00272087

6. Cunningham TP, Montelaro RC, Rushlow KE. Lentivirus envelope sequences and pro viral genomes are stabilized in *Escherichia coli* when cloned in low-copy-number plasmid vectors. Gene. 1993;124: 93–98. doi:10.1016/0378-1119(93)90766-V

7. Bolivar F, Rodriguez RL, Greene PJ, Betlach MC, Heyneker HL, Boyer HW, et al. Construction and characterization of new cloning vehicles. II. A multipurpose cloning system. Gene. 1977;2: 95–113.

8. Datsenko KA, Wanner BL. One-step inactivation of chromosomal genes in *Escherichia coli* K-12 using PCR products. Proc Natl Acad Sci USA. 2000;97: 6640–6645. doi:10.1073/pnas.120163297

9. Uzzau S, Figueroa-Bossi N, Rubino S, Bossi L. Epitope tagging of chromosomal genes in *Salmonella*. Proc Natl Acad Sci USA. 2001;98: 15264–15269. doi:10.1073/pnas.261348198

10. Ellermeier CD, Janakiraman A, Slauch JM. Construction of targeted single copy lac fusions using lambda Red and FLP-mediated site-specific recombination in bacteria. Gene. 2002;290: 153–161.

11. Birmingham CL, Smith AC, Bakowski MA, Yoshimori T, Brumell JH. Autophagy controls *Salmonella* infection in response to damage to the Salmonella-containing vacuole. J Biol Chem. 2006;281: 11374–11383. doi:10.1074/jbc.M509157200
