# Supplementary material for: Expression of a novel class of bacterial Ig-like proteins is required for IncHI plasmid conjugation
Source: PLoS Genet. 2019 Sep 17;15(9):e1008399. doi: 10.1371/journal.pgen.1008399 (PMC6764697; doi:10.1371/journal.pgen.1008399)
Supplement: S3 Table — (DOCX) [file pgen.1008399.s010.docx]

**S3 Table.**

| **Name** | **Sequence 5’ - 3’** | **Use** |
| --- | --- | --- |
| RS0009_P1 | TTAATTGCTTCGATTAAATCGGTACAAGCTGAGGCAATTGTGTAGGCTGGAGCTGCTTC | *rsp* deletion |
| RS0009_P2 | GGACATTTCTGCCCCGGCTGTTATTTCATTAGCTTGCCCATATGAATATCCTCCTTAGT | *rsp* deletion |
| RS0009_up_for | ACAGGTGTTCGTCATGAGAA | Confirmation RSP deletion |
| RS0009_down_rev | CTTTCCCGACGTTACCCCAT | Confirmation RSP deletion |
| LacZ_R | GATGACCTGCAAGGCGATTA | Confirmation *lacZ* insertion |
| CatC1 | TTATACGCAAGGCGACAAGG | Confirmation of Cm^R^ insertion |
| 09-EcoRI-pLG_for | CCGGAATTCCATCTGAGTCTAAGAGCGGTCAGTGAG | RSP cloning pLG338-30 |
| 09-BamHI-pLGR | CGGGATCCGTTTCATTAGCTTGCCTTACTGCGAGGTTT | RSP cloning pLG338-30 |
| R27_p0103XP1 | GGGCGCGTCAAAGTCCGTAGATTCAGTTGAAACCTCGCAGGACTACAAAGACCATGACGG | RSP-Flag |
| R27_p0103XP2 | TTTCTGCCCCGGCTGTTATTTCATTAGCTTGCCTTACTGCCATATGAATATCCTCCTTAG | RSP-Flag |
| R27_p0103XP1UP | GGTATATCAATGATGAGCTAAGG | RSP-Flag confirmation |
| R27_p0103XP2DOWN | GTGATTGTCCTGGCCGCTGTC | RSP-Flag confirmation |
| trhCP1 | GTCAAACGGTATTGTGAGACAACTCTGAGGTCACGAATGAGAGTGTAGGCTGGAGCTGCTTC | *trhC* deletion |
| trhCP2 | GTCAAAACAAAATGAGGTAACCAGTAACTTAAAACGTCAGGCCATATGAATATCCTCCTTAGT | *trhC* deletion |
| trhCP1UP | GGAAGTCCAGGATGCGGCTCGTG | *trhC* deletion confirmation |
| trhCP2DOWN | CTTAACCCGGGCTATTATTTC | *trhC* deletion confirmation |
| qRS0009FW | TTTGCCTGTCACGCTGCTGG | qRT-PCR |
| qRS0009RV | CGGGCGTAAATGGTCGGAGG | qRT-PCR |
| RSPBig3_31FW | AGAAGGAGATATAACTATGTATCTGTATATCTTTGACTTAACAGACTTG | Cloning Big3_·3 domain |
| RSPBig3_31RV | GTGGTGGTGATGGTGATGGCCTGGTTGATACGTGAAAGTAGTATTAAATGT | Cloning Big3_·3 domain |
| SL1344_flgEP1 | AAATAATCTAAGCCCTTACACTTATCAGGAGTCAGTCATGTCTGTGTAGGCTGGAGCTGCTTC | *flgE* deletion |
| SL1344_flgEP2 | AAATTGCGTGATCCATTAAGCTATCCCGTCAGGCGCTTAGCGCATATGAATATCCTCCTTAGT | *flgE* deletion |
| SL1344_flgEP1Up.1 | GGTACAGGGCGTGACGAAGG | *flgE* deletion confirmation |
| SL1344_flgEP2Down.1 | CATTAGCCAGGTTGCTGGCCG | *flgE* deletion confirmation |
| gapAFW | TTTCCGTGCTGCTCAGAAAC | qRT-PCR |
| gapARV | TTTCCGTGCTGCTCAGAAAC | qRT-PCR |
| trhCBamHIFW | CGGGATCCTTGAAAAAGTTACTTTTGTCT | *trhC* cloning pBR322 |
| trhCBAMHIRV | CGGGATCCTCAGGCAGCTGATTTGCTCGC | *trhC* cloning pBR322 |
